# A Novel Role of CDX1 in Embryonic Epicardial Development

**DOI:** 10.1371/journal.pone.0103271

**Published:** 2014-07-28

**Authors:** Min Chu, Libo Wang, Huan Wang, Ting Shen, Yanqin Yang, Yun Sun, Nannan Tang, Ting Ni, Jun Zhu, Richard B. Mailman, Yuan Wang

**Affiliations:** 1 Shanghai Key Laboratory of Regulatory Biology, Institute of Biomedical Sciences and School of Life Sciences, East China Normal University, Shanghai, China; 2 State Key Laboratory of Genetics Engineering & MOE Key Laboratory of Contemporary Anthropology, School of Life Sciences, Fudan University, Shanghai, China; 3 Systems Biology Center, National Heart, Lung, and Blood Institute, National Institutes of Health, Department of Health and Human Services, Bethesda, Maryland, United States of America; 4 Department of Pharmacology, Penn State College of Medicine, Hershey, Pennsylvania, United States of America; William Harvey Research Institute, Barts and The London School of Medicine and Dentistry, Queen Mary University of London, United Kingdom

## Abstract

The molecular mechanism that regulates epicardial development has yet to be understood. In this study, we explored the function of CDX1, a *Caudal*-related family member, in epicardial epithelial-to-mesenchymal transition (EMT) and in the migration and the differentiation of epicardium-derived progenitors into vascular smooth muscle cells. We detected a transient expression of CDX1 in murine embryonic hearts at 11.5 days post coitum (dpc). Using a doxycycline-inducible CDX1 mouse model, primary epicardium, and *ex vivo* heart culture, we further demonstrated that ectopic expression of CDX1 promoted epicardial EMT. In addition, a low-dose CDX1 induction led to enhanced migration and differentiation of epicardium-derived cells into α-SMA+ vascular smooth muscles. In contrast, either continued high-level induction of CDX1 or CDX1 deficiency attenuated the ability of epicardium-derived cells to migrate and to mature into smooth muscles induced by TGF-β1. Further RNA-seq analyses showed that CDX1 induction altered the transcript levels of genes involved in neuronal development, angiogenesis, and cell adhesions required for EMT. Our data have revealed a previously undefined role of CDX1 during epicardial development, and suggest that transient expression of CDX1 promotes epicardial EMT, whereas subsequent down-regulation of CDX1 after 11.5 dpc in mice is necessary for further subepicardial invasion of EPDCs and contribution to coronary vascular endothelium or smooth muscle cells.

## Introduction

The formation of heart is a complicated morphogenetic process that requires participation of cells from different embryonic origins [1]. The epicardial cells which cover both the heart and the intrapericardial part of the great arteries are originally derived from the proepicardium with different developmental potentials [2], [3]. In mice around 11.5 days post coitum (dpc), epicardium undergoes EMT to become developmentally plastic mesenchymal cells [2], [3]. These epicardium-derived cells (EPDCs) then invade into subepicardial layers and further generate cardiac fibroblasts, vascular smooth muscles, and a fraction of coronary endothelial cells [2], [3]. Disturbance of epicardial EMT may lead to congenital heart diseases including non-compaction cardiomyopathy, deficient annulus fibrosis, valve malformations and coronary vascular abnormalities [2], [3]. In addition, recent evidence suggests that reactivation of cardiac epicardium contributes to the repair processes in diseases such as myocardial infarction [2], [3]. Therefore, research on epicardial EMT regulation may offer novel therapeutic strategies to prevent or to treat heart diseases.

During EMT, the cell-to-cell junctions of epicardium are dissembled, and the expression of intercellular adhesion molecule E-cadherin is downregulated with induction of Snail proteins [2], [3]. In addition, epicardial EMT is regulated by multiple transcription factors including TCF21, Wilms' Tumor 1 (WT1), NFATC1, and TBX18,as well as signaling molecules such as TGF-β1, BMPs, retinoic acid (RA), FGF, and NOTCH [2], [3]. Among them, *Wt1* encodes a zinc-finger protein that is specifically expressed in proepicardium and epicardium, but not in myocardium, and thus has been used as a marker for epicardium [4]. To date the molecular interplays among specific signaling pathways and transcription factors that regulate epicardial EMT and EPDC differentiation into particular cell lineages, including vascular smooth muscles and coronary endothelial cells, have yet to be well characterized.

The CDX family members (CDX1, CDX2, and CDX4) are *Caudal*-related transcription factors and have been originally implicated in anterior-posterior patterning during embryonic development [5]. All *Cdx* genes can be detected in the posterior primitive streak of mouse embryos around 7 dpc and display a posterior-to-anterior concentration gradient during late embryogenesis. Consistent with this expression pattern, it has been demonstrated that CDX proteins played important roles in defining the posterior identity of various tissues. For example, loss of function analyses show that CDX family members function redundantly as upstream regulators for *HOX* genes to participate in axial elongation, blood formation, placenta vascular construction, intestinal specification, and neurological development [6], [7], [8], [9], [10], [11]. The defects caused by *CDX* null mutations cannot, however, be entirely explained by their actions on *HOX* genes. Several non-*HOX* targets of CDX family members have been identified, including *Sall4* (in hematopoietic specification) [12], *Wnt3a*, *Cyp26a1* and *T* (*Brachyury*, in posterior axis formation) [13], and *p21/Waf* (in colon carcinogenesis) [14]. The identification of novel CDX targets may thus help us to illuminate the physiological and pathological functions of CDX family members in tissue development and organ formation.

Recently, Lengerke *et al*. demonstrated that CDX proteins suppressed cardiac differentiation from mouse embryonic stem cells (ESCs) and in zebrafish [15], but the underlying molecular mechanism of this phenomenon has not been thoroughly understood. In the current study, we find that CDX1 is transiently expressed in embryonic hearts at 11.5 dpc. In addition, we observed that high-level induction of CDX1 promoted epicardial EMT, but blocked migration of EPDCs and impaired formation of coronary vascular structure. Additionally, data from RNA-seq analyses imply that high-level expression of CDX1 may disturb the differentiation of EPDCs by upregulating genes in neuronal development and by downregulating genes involved in vascular endothelial growth,thereby providing novel insights into the molecular mechanisms of CDX1 in regulating cardiac development.

## Materials and Methods

### Ethics statement

All animal experimental procedures were conducted in accordance with the Animal Welfare Act and Public Health Service Policy in China and consistent with the WMA Statement on Animal Use in Biomedical Research. The protocol was approved by the Committee on the Ethics of Animal Experiments at East China Normal University. All animals were euthanized by carbon dioxide inhalation followed by cervical dislocation before dissection of heart tissues.

### Generation of doxycycline-inducible CDX1 ESC lines and transgenic mice

The protocols for constructing doxycycline-inducible ESC line and transgenic mice have been previously described [16], [17]. Specifically, to establish doxycline inducible CDX1 ESC lines, a fragment containing *Cdx1* cDNA was cloned into the *EcoRI* site of pBS31RBGpA and was electroporated with plox-Fle into KH2 parental mouse ESCs (a kind gift from Dr. Rudolf Jaenisch). Resistance of hygromycin by ESC colonies indicates a correct insertion of *Cdx1* cDNA at downstream of the collagen 1a locus by frt/Flpase-mediated site-specific integration. Individual colonies were picked and confirmed for CDX1 induction by doxycycline. A correctly targeted ESC line was maintained, expanded, and injected into blastocysts derived from C57BL/6 female mice. We designated mice with a M2 reverse tetracycline transactivator at the ROSA26 locus from one allele as *rtTA^−/+^* (*rtTA^+/+^* for transactivator at both alleles), while mice with a doxycline inducible *Cdx1* transgene were named *Cdx1^Tg^*. For CDX1 induction in embryos, pregnant mice were fed with 10 µg/ml doxycycline in the drinking water containing 10 mg/ml sucrose. The age of embryo was defined as 0.5 dpc at noon on the day of vaginal plug observation. Primers for genotyping were provided in Table S1.

### Karyotyping

To examine the chromosomal integrity of CDX1 targeted cells, ESCs were incubated with 0.1 µg/ml colcemid for 2 h at 37°C, washed twice with PBS, disaggregated with 0.25% trypsin, and then resuspended in 75 mM KCl at room temperature for 15 minutes. The cells were fixed in ice-cold fixative (methanol:glacial acetic acid, 3∶1 freshly prepared) and spread onto a pre-cleaned glass slide. The air-dried slides were examined under a microscope after staining with 2% Giemsa. At least 15 cells at metaphase were analyzed for the number and quality per chromosome spread.

### Primary culture of epicardium and induction of α-SMA+ cells from EPDCs

Embryonic hearts at 11.5 dpc were harvested and placed onto gelatin-coated tissue culture dishes containing pre-warmed medium (DMEM with 10% fetal bovine serum, and 2 mM L-Glutamine, all from Invitrogen) and incubated at 37°C. Heart clumps were removed after 48 h. When growing confluence, the remaining cobblestone-like epicardial cells were passed onto new gelatin-coated plates for further assays.

### Wound scratch/migration assay

For wound scratch/migration assay, the primary epicardial cells at 80% confluence were serum-starved for 12 h and a straight line or a cross was introduced with a pipette tip. Cells were gently washed twice with medium and allowed to grow for 12 h or 24 h before photography.

### BrdU incorporation assay

For this assay, primary epicardial epithelial cells on coverslips were incubated with 10 mM BrdU for 30 minutes at 37°C. Cells were washed with PBS three times, fixed in 4% paraformaldehyde for 15 minutes at room temperature, and permealized with 0.1% Triton X-100. Incubation of FITC-labeled antibody against BrdU overnight at 4°C was followed. Cells were finally washed with PBS and stained with DAPI for photography with Leica Microsystems (Model DM4000B, Wetzlar, Germany)

### RNA interference and RT-PCR

Scrambled siRNAs or siRNAs against *CDX1* (Santa Cruz Biotechnology) were transfected at a final concentration of 10 nM using HiPerFect Transfection Reagent (Qiagen). Cells were collected for RNA preparation or fixed in 4% paraformaldehyde (PFA) for immunofluorescent analyses. Total RNA was extracted using Trizol (Invitrogen) and quantitative PCR was performed on Mx3005P (Stratagene). Transcript levels were normalized to GAPDH. Primers for RT-PCR are listed in Table S1. All real-time PCR data are presented as average of 4–6 replicates ±1 standard error of the mean (s.e.m.) from 2-3 independent experiments.

### RNA-seq assays and data analysis

Total RNAs were collected from epicardium of Cdx1^Tg^rtTA^-/+^ embryos at 11.5 dpc treated with or without 1 µg/ml doxycycline for two days. 2 µg of DNA-free total RNA was then applied for two rounds of polyA+ RNA selection. The resulting RNA was subjected for dUTP mediated RNA-seq library construction. We performed 14 cycles of PCR using Phusion Hot Start High-Fidelity DNA Polymerase (Finnzymes) to generate the final library. Strand-specific RNA-seq library was constructed according to published protocols [18]. For each sample more than 200 million paired 50mer reads were obtained using Illumina Hiseq2000 platform. The raw reads were mapped to the reference mouse genome (mm10) by Tophat (v2.0.8b) (http://tophat.cbcb.umd.edu/index.shtml). Reads that aligned equally well to two or more locations in the reference genome were discarded. The read count and RPKM (Reads Per Kilobase per Million) of mRNA transcripts level were calculated using RSeQC (http://rseqc.sourceforge.net/) based on mm10 RefSeq gene model. The transcripts with read count less than 10 in both samples were filtered out. Differentially expressed transcripts were selected between two conditions. The original RNA-seq raw data were uploaded to Sequence Reads Archive (SRA) under an accession # as SRP041744 (http://www.ncbi.nlm.nih.gov/sra/?term=SRP041744).

### Heart explants and CMFDA labeling assay

For *ex-vivo* culture, hearts at 11.5 dpc were dissected and cultured at 37°C on a shaker. For CMFDA labeling assay, the explanted hearts were treated with doxycycline for 6 h and incubated with 10 µM CMFDA (Invitrogen) at 37°C for 15 min. Hearts were washed twice, and incubated in DMEM containing 10% FBS with different dose of doxycycline for 48 h before histological analyses.

### Immunofluorescence (IF), Histology and Immunohistofluorescence (IHF)

IF and hematoxylin/eosin histological analyses were performed following a published protocol [19]. All IHF was performed on 4 µm frozen sections. Primary antibodies used in these assays: CDX1 (1: 400, Novus), WT1 (1∶50, Santa Cruz Biotechnology), and c-TNNT2 (1∶50, Hybridoma), β-catenin (1∶500, Epitomics), or α-SMA (1∶400, Abcam). Cardiac sections or cells were incubated with anti-mouse and anti-rabbit secondary antibodies conjugated to FITC or TRITC (1∶500 dilution, Jackson lab) for photography with Leica Microsystems (Model DM4000B,. Wetzlar, Germany).

### Whole-mount immunohistochemistry (WIHC)

WIHC was performed on hearts at 14.5 dpc according to standard protocols [4]. Briefly, after fixation, dehydration, bleach, and rehydration, hearts were incubated with an antibody against PECAM (1∶50, BD Biosciences) at 4°C. DAB detection kit was used for color development and photographed with an Olympus Dissecting Microscope.

### Statistical analysis

All data were presented as mean ±1 s.e.m. *p* values were calculated from Student's t-test for comparisons when indicated.

## Results

### CDX1 is highly expressed in hearts at 11.5 dpc

**Figure 1:**
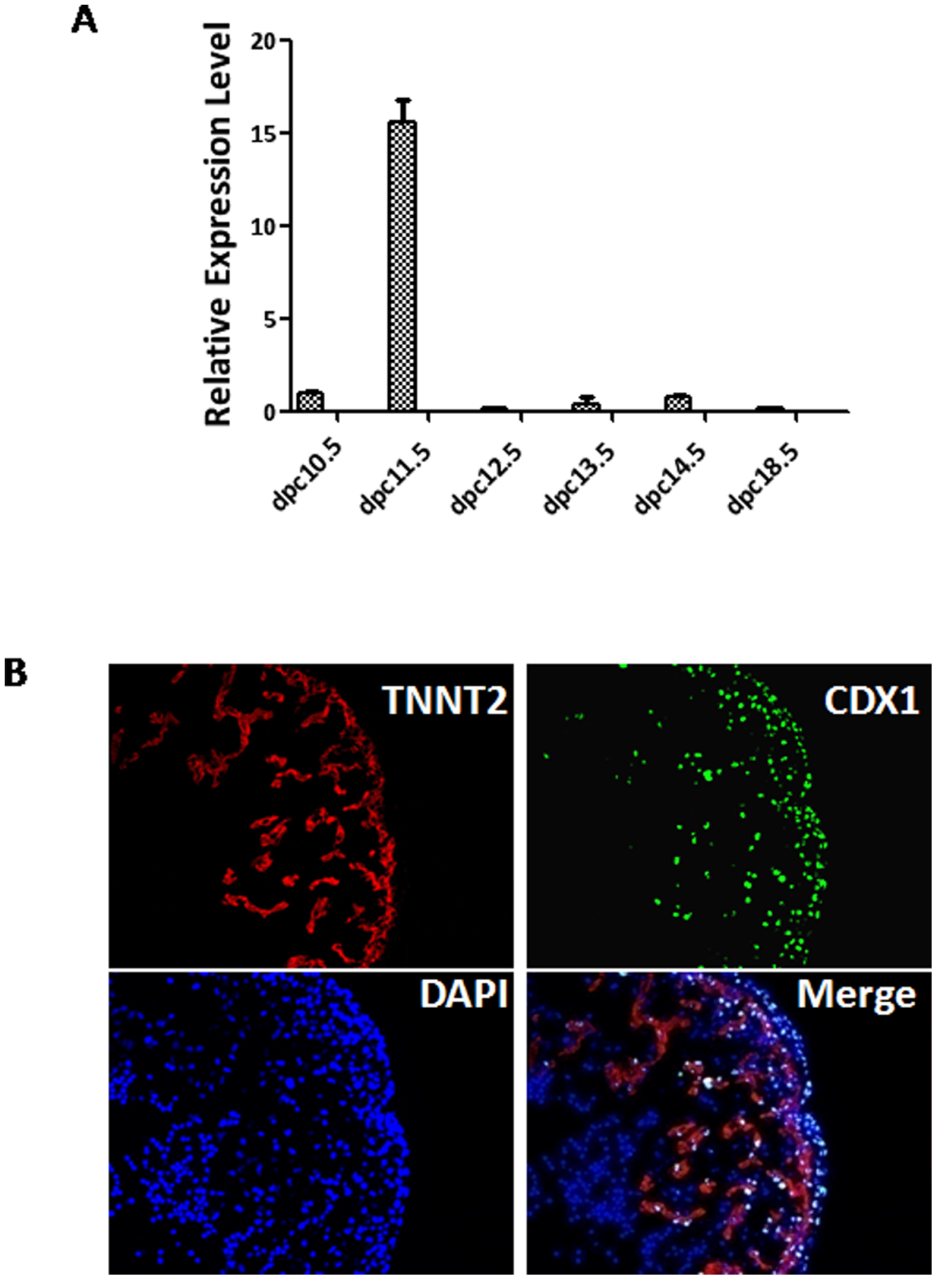
Transient expression of CDX1 in embryonic hearts at 11.5 dpc. (A) Transcript levels of CDX1 measured by real-time RT-PCR in hearts collected at different time points during embryonic development. (B) IF of CDX1 and c-TNNT in hearts collected at 11.5 dpc.

It was recently reported that ectopic CDX1 expression inhibited cardiomyocyte formation from mESCs and zebrafish, suggesting its role in cardiac development [15]. To understand its potential function *in vivo*, we isolated hearts at different time points during embryonic development and measured the transcript levels of *Cdx1*. Interestingly, we observed a transient peak expression of *Cdx1* in hearts at 11.5 dpc (Figure 1A). We further dissected embryonic hearts at 11.5 dpc and examined CDX1 expression by immunofluorescence assays. CDX1 proteins were indeed detected in the cardiomyocytes co-stained with c-TNNT2 (Figure 1B). More importantly, CDX1 expression was also observed in epicardium covering the heart (Figure 1B). These data suggest that CDX1 may play a role during embryonic cardiac development.

### Enforced expression of CDX1 leads to coronary vascular abnormalities

**Figure 2:**
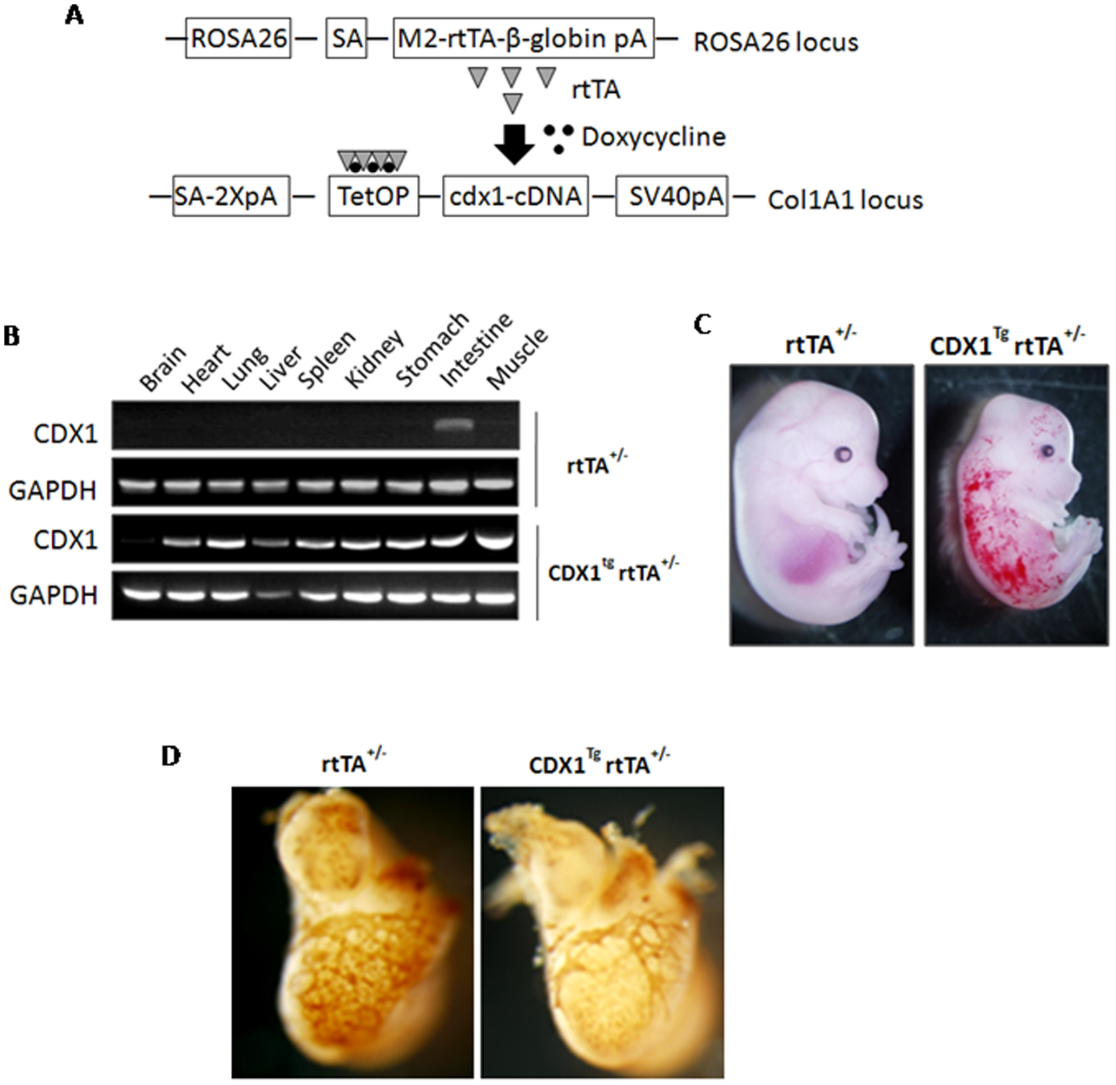
Ectopic expression of CDX1 affects embryonic heart development. (A) Strategy to establish doxycycline-inducible CDX1 transgenic ESCs and mice. ESCs contains a M2 reverse tetracycline transactivator at the ROSA26 locus, and a CDX1 cDNA driven by doxycycline-responsible element was integrated at downstream of the collagen 1a locus by frt/Flpase-mediated site-specific integration. (B) The expression of *CDX1* among various adult tissues from mice upon 0.5 mg/ml doxycycline treatment in drinking water for two days. (C) Morphology of embryos collected at 14.5 dpc. n = 10. (D) WIHF with an antibody against PECAM on hearts isolated at 14.5 dpc. n>5. (C–D) Embryos were collected from pregnant mice treated with 10 µg/ml doxycycline in drinking water from 11.5 dpc to 14.5 dpc. n>10.

To explore the role of CDX1 in cardiac development during embryogenesis, we examined the effects of ectopic expression of CDX1. We utilized an ESC line expressing the M2 reverse tetracycline transactivator (*M2-rtTA*) from ROSA26 locus [16]. A *Cdx1* cDNA driven by a doxycycline responsive element was targeted downstream of collagen 1a by frt/Flpase-mediated site specific integration (Figure 2A). In this way, ESC lines were established in which CDX1 expression could be induced upon doxycycline treatment (Figure S1A). We further obtained a *Cdx1* transgenic mouse model with correctly targeted ESCs by blastocyst injection. Exposing these mice to doxycycline in drinking water led to a strong upregulation of *Cdx1* in almost all tissues except brain (Figure 2B), probably due to the inability of doxycycline to cross the brain-blood barrier. In further experiments, *Cdx1^Tg^rtTA^+/+^* male mice were crossed with wild-type C57BL/6 female mice to get *rtTA^-/+^* (non-inducible ones without *Cdx1* transgene as littermate controls) and *Cdx1^Tg^rtTA^-/+^* (inducible CDX1 upon doxycycline treatment) embryos, orgnas, or epicardial cells. Doxycycline was used in both non-inducible controls and inducible hearts or epicardium in all experiments to exclude the bias caused by the any effects of doxycycline on cardiac development.

As peak expression of CDX1 was detected in embryonic hearts, we induced CDX1 by adding 10 µg/ml doxycycline to the drinking water for pregnant mice at 11.5 dpc and collected embryos after 72 h. Embryos at 14.5 dpc under CDX1 induction were slightly smaller compared to their littermate controls, and displayed accumulation of blood in systemic veins (Figure 2C). We further dissected hearts, lungs, kidneys/gonads, and liver from these embryos, and found that the gross morphology and size of these organs were normal (Figure S2A). However, using whole-mount immunohistochemistry of PECAM, a marker for vascular endothelial cells, we found that the coronary arteries failed to form properly in CDX1-induced embryos (Figure 2D). These findings are reminiscent of the *Wt1* knockout mouse model that displays a defect in epicardial EMT [4], and thus implicate a role of CDX1 in epicardial development during embryogenesis. By contrast, no obvious difference was detected in the gross morphology or PECAM staining between *rtTA^−/+^* and *Cdx1^Tg^rtT^−/+^* embryos without treatment of doxycycline (Figure S2B–D), suggesting that the phenotype we observed was not due to low-dose leakage of CDX1.

### Ectopic expression of CDX1 promotes epicardial EMT and EPDC formation

**Figure 3:**
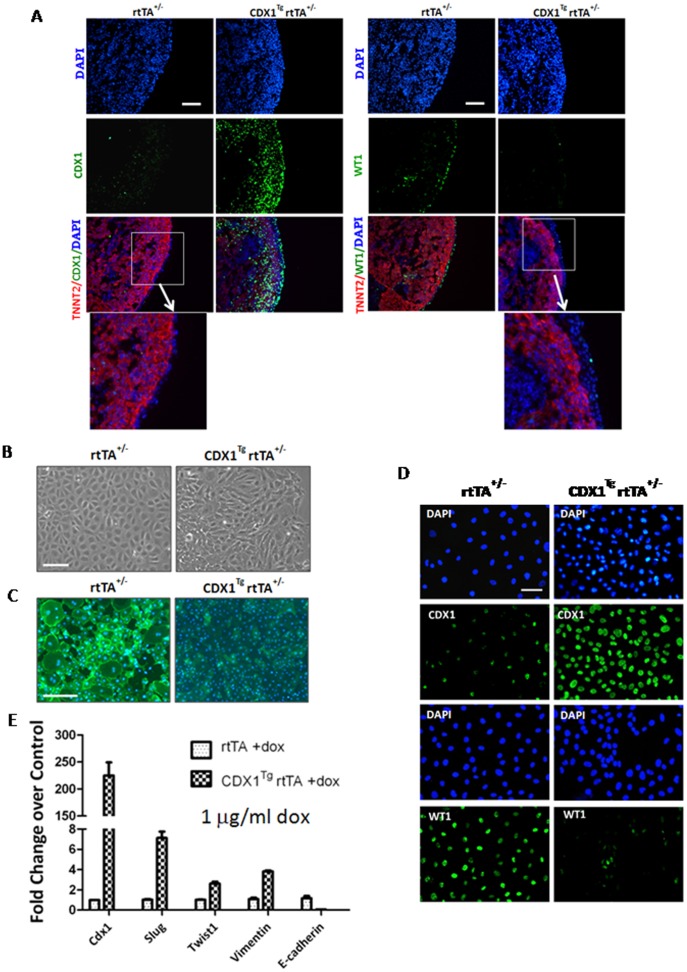
Ectopic expression of CDX1 promotes epicardial EMT. (A) IHF with antibodies against CDX1, c-TNNT2 or WT1 on cardiac sections from embryos at 14.5 dpc. Lower panels show images with higher magnification. Scale bars: 120 µm. Embryos were collected from pregnant mice treated with 10 µg/ml doxycycline in drinking water starting from 11.5 dpc. n>5. (B) Morphology of control epicardium or epicardium upon CDX1 induction by doxycycline treatment. (C) Staining of β-catenin by IF on control epicardium or epicardium upon CDX1 induction in the presence of doxycycline. (B–C): Scale bars: 400 µm. (D) IF with antibodies against CDX1 and WT1 on primary epicardium collected from embryos at 11.5 dpc in the presence of 1 µg/ml doxycycline for 48 h. Scale bars: 90 µm. (E) Real-time RT-PCR on *CDX1* and genes involved EMT on samples described in (D). Doxycycline at 1 µg/ml was used in both non-inducible controls and inducible hearts or epicardium in all experiments to exclude the bias caused by the any effects of doxycycline on cardiac development.

As CDX1 is highly expressed in epicardium at 11.5 dpc, a time window during which epicardial EMT occurs [2], [3], [20], and the defects of transgenic embryos upon CDX1 induction mimics *Wt1* null mutants [4], we next investigated whether CDX1 participated in epicardial function. We performed whole heart *ex vivo* culture from transgenic embryos at 11.5 dpc and induced CDX1 overexpression with doxycycline treatment. After CDX1 induction for 48 h by 1 µg/ml doxycycline, we observed that the epicardial outlayers (the c-TNNT2 negative population) covering heart became thicker as manifested by co-staining with c-TNNT2 and DAPI (Figure 3A). In addition, these cells lost the expression of the epicardium marker WT1 upon ectopic CDX1 induction (Figure 3A), suggesting CDX1 indeed affects epicardial development.

To further define the role of CDX1 in epicardium, we established primary epicardial culture isolated from embryos at 11.5 dpc. The non-inducible *rtTA^+/−^*cells displayed a cornerstone epithelial morphology with intense staining of β-catenin, especially at sites of cell-to-cell contact (Figure 3B–C), and WT1 (Figure 3D, left bottom panel), proving that we had successfully established primary culture for epicardium. When subjected to CDX1 induction by doxycycline, these inducible *Cdx1TgrtTA^+/−^* cells adapted a spread mesenchymal morphology and lost the positivity for β-catenin and WT1 (Figure 3B–D). By contrast, no obvious changes in epicardial outlayers or WT1 staining were observed in hearts or epicardium which were collected from *rtTA^−/+^* and *Cdx1^Tg^rtTA^−/+^*embryos and cultured in the absence of doxycycline (Figure S4A& S4D ), indicating that low-dose leakage of CDX1 did not affect epicardial development. Additionally, quantitative gene expression analyses by real-time RT-PCR demonstrated that the CDX1 induction led to reduced transcript level of *E-cadherin*, and upregulated expression of *Slug, Twist1*,and *Vimentin*, genes that are important for EMT (Figure 3E). Taken together, data from both *in vitro* assays and the explanted hearts support a role of CDX1 in promoting EMT of epicardium into WT1 and β-catenin negative EPDCs.

### CDX1 affects the migration and the differentiation of EPDCs into vascular smooth muscles

**Figure 4:**
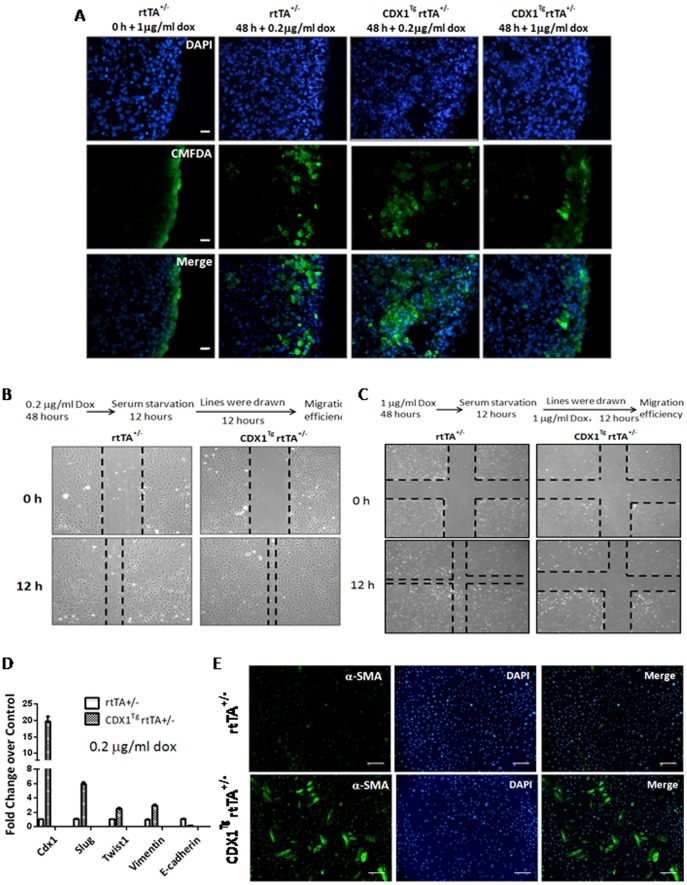
Ectopic expression of CDX1 affects the migration and the differentiation of EPDCs. (A) Invasion of CFMDA-labeled EPDCs into subepicardial layer of explanted hearts collected at 11.5 dpc was monitored at 0 or 48 h post labeling in the presence of doxycycline. Scale bars: 33 µm. (B–C) Wound scratch assays were performed on primary control epicardium or epicardium upon CDX1 induction following the different work regimens above the panel. (D) Real-time RT-PCR on primary cultured control epicardium or epicardium upon CDX1 induction with 0.2 µg/ml doxycycline for 48 h. (E) Immunofluorescence assays with an antibody against α-SMA on primary control EPDCs or EPDCs upon CDX1 induction with 0.2 µg/ml doxycycline. Scale bars: 400 µm.

Previous studies demonstrated that EPDCs could further migrate into subepicardial regions and contribute to various types of cells [2], [3], [20]. As CDX1 appeared to promote epicardial EMT and EPDC formation from epicardium *in vitro* and from explanted hearts, we next investigated the role of CDX1 in migration of EPDC by selective dye-labeling of epicardium. The explanted hearts collected at 11.5 dpc were pre-treated with different doses of doxycycline for 6 h, followed by a brief incubation of CMFDA in culture media, and then placed into dye-free media in the presence of doxycycline for 48 h. Consistent with published results, CMFDA only labeled epicardium under this condition as showed by heart dissection immediately after the withdrawal of CMFDA treatment (Figure 4A). Upon CDX1 induction with 0.2 µg/ml doxycycline, more labeled epicardium-derived population invaded into subepicardial regions, whereas high-dose induction of CDX1 by 1 µg/ml doxycycline resulted in accumulation of CFMDA-labeled EPDCs at the outlayers of hearts (Figure 4A & Figure S4E). Taken together, these data suggest that EPDC migration is promoted by low-level CDX1 expression but is inhibited by high-level CDX1 induction.

We next used wound scratching assays to determine the migration efficiency of the primary epicardium isolated at 11.5 dpc upon different regimens of CDX1 induction. In these assays, gaps with same space were introduced in control and doxycycline-inducible cells after serum starvation, whereupon migration was monitored. We clearly observed that in the group with CDX1 induction by doxycycline at 0.2 µg/ml, more EPDCs filled the gap where cells were previously removed (Figure 4B & Figure S5C). By contrast, high-dose CDX1 induction by doxycycline at 1 µg/ml significantly inhibited the migration of EPDCs (Figure 4C & Figure S5C). In addition, we confirmed that *Cdx1* induction is dose-dependent on doxycycline concentration (Figure S5F). These data are thus in agreement with our observation from explanted hearts that the CDX1 affects the migration of EPDCs in a dose-dependent manner. Furthermore, we found that low-dose treatment of doxycycline at 0.2 µg/ml caused similar changes in expression of genes in EMT as occurred upon high-level doxycycline induction (Figure 3E & Figure 4D). Therefore, it is unlikely that the difference of migration efficiency in EPDCs is caused by distinct EMT levels.

Ectopic CDX1 expression *in vivo* affected the formation of coronary arteries (Figure 2D), so we next explored the potential roles of CDX1 in the differentiation of EPDCs into vascular smooth muscles. In our system, only a small fraction of primarily cultured epicardium could spontaneously differentiate into α-SMA+ smooth muscles after culture for more than four days. Interestingly, the percentage of these α-SMA+ vascular smooth muscle cells increased significantly upon CDX1 induction with 0.2 µg/ml doxycycline (Figure 4E & Figure S5D), implicating a role of CDX1 in the formation of coronary arteries from EPDCs.

### Depletion of CDX1 blocked the migration and the differentiation of EPDCs

**Figure 5:**
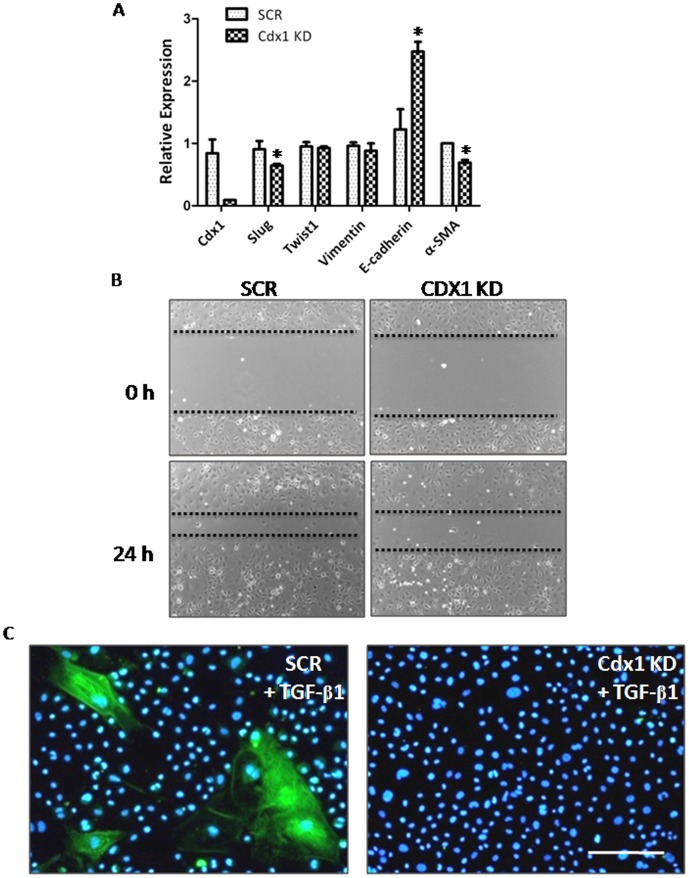
CDX1 depletion blocks the migration and the differentiation of EPDCs. (A) Wound scratch assay performed on primary control epicardium (SCR; with scrambled siRNA) or epicardium upon depletion of CDX1 (CDX1 KD). (B) Real-time RT-PCR analyses on samples described in (A). * *p*<0.05. (C) Immunofluorescence assays with an antibody against α-SMA on control EPDCs or EPDCs upon CDX1 depletion in the presence of 5 ng/ml TGF-β1. Scale bars: 400 µm.

To ascertain the physiological function of CDX1 in cardiac development, we knocked down CDX1 in primarily cultured epicardium (Figure 5A). We found that the expression of *E-cadherin* was significantly increased, whereas *Slug* level was modestly downregulated (Figure 5A), suggesting that EMT was blocked upon CDX1 depletion. Subsequently, we observed that CDX1 knockdown also led to reduced migration of EPDCs in wound scratching assays (Figure 5B & Figure S5C).

It was previously reported that TGF-β1 could induce the differentiation of EPDCs into coronary smooth muscle cells [21]. We thus examined if CDX1 played a role in this process. Primary epicardium isolated at 11.5 dpc was treated with 5 ng/ml of TGF-β1 in the presence of scrambled control siRNAs or siRNAs against CDX1, and was cultured for four more days to allow the differentiation of EPDCs. Compared to scrambled siRNA control, CDX1 depletion markedly blocked the maturation of EPDCs into α-SMA+ vascular smooth muscle cells induced by TGF-β1 (Figure 5C). These data thus implicate that CDX1 not only affects epicardial EMT, but also plays a role in the migration and the differentiation of EPDCs induced by TGF-β1 signaling.

### Ectopic CDX1 expression affects genes in neuronal development, vascular endothelial growth, and cell adhesion

**Figure 6:**
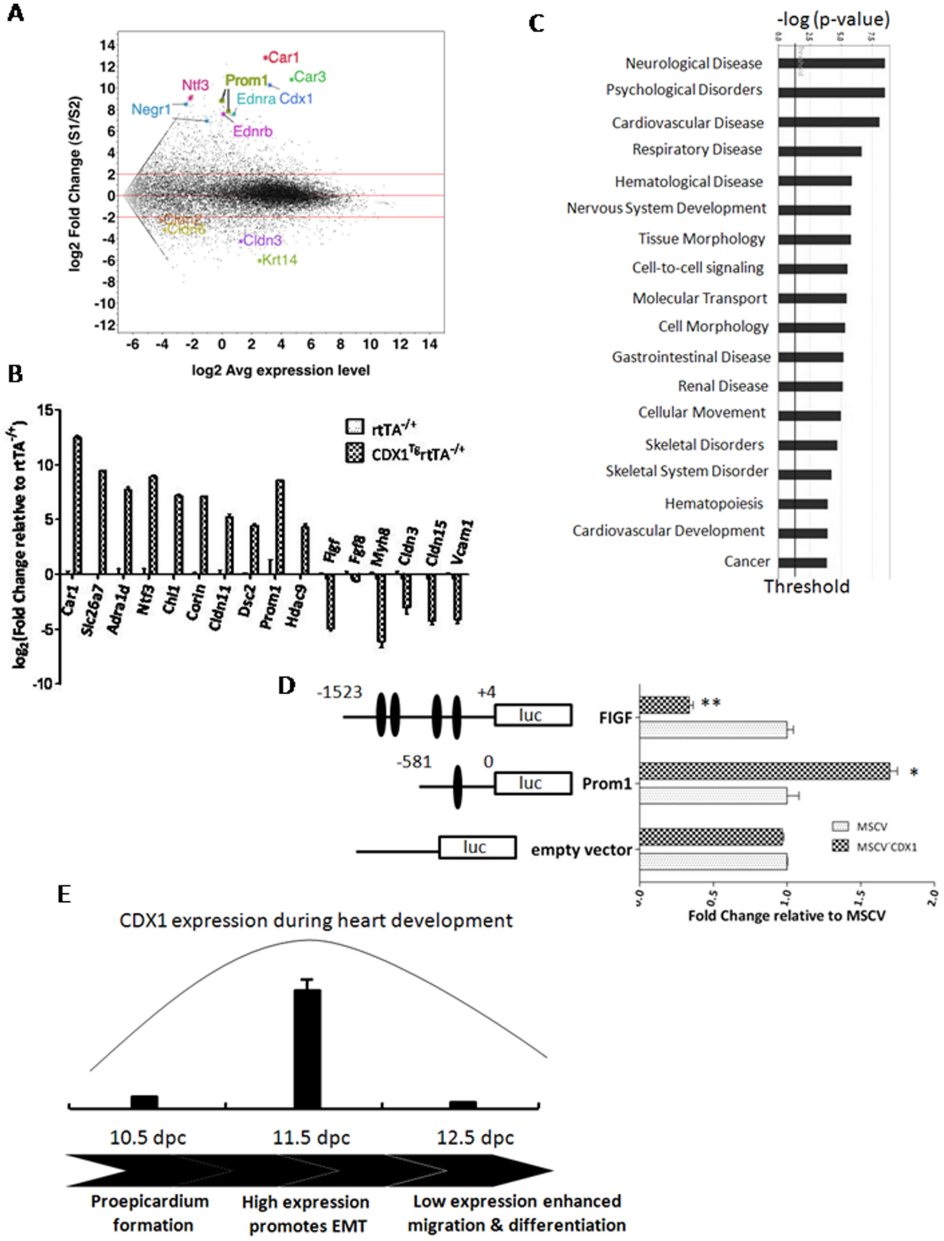
Ectopic expression of CDX1 affects the transcript levels of genes that are important for neuronal development, vascular endothelial growth, and cell adhesions. (A) Differentially expressed genes identified by RNA-seq upon ectopic induction of CDX1 with 1 µg/ml of doxycycline for two days. For each annotated transcript, the expression fold change (y-axis) between the two conditions (control *vs* CDX1 induced) was plotted against the geometric mean of their expression level (x-axis). A subset of transcripts involved in neuronal regulation and cell adhesion are highlighted. (B) Real-time RT-PCR analyses on primary cultured control epicardium or epicardium upon CDX1 induction with 1 µg/ml doxycycline for 48 h. (C) Genes affected upon CDX1 induction in epicardium were analyzed by IPA according to their functional categories. (D) Luciferase reporter activity was measured in 293T cells co-transfected with a luciferase/renilla dual reporters and a control empty vector (MSCV) or a CDX1 expressing plasmid (MSCV-CDX1). Luciferase expression was driven by various promoters containing CDX1 binding sites (in closed ovals). Data were presented as the average of triplicates in luciferase activity (relative to renilla) ± one s.e.m from a representative experiment. Data were reproducible from two other independent experiments. *: *p*<0.05; **: *p*<0.01. (E) Model of function for CDX1 during embryonic epicardial development. Peak expression of CDX1 in hearts occurs at 11.5 dpc and promotes EMT of epicardium. Reduced level of CDX1 after 11.5 dpc allows further migration and the differentiation of EPDCs into vascular components.

In order to elucidate the molecular mechanism by which CDX1 affects epicardial function, we performed genome-wide expression analyses by RNA-seq on mouse primary epicardium isolated at 11.5 dpc. Compared to non-induced control cells, the expression level of 704 genes was altered by more than a 16-fold change upon ectopic CDX1 induction at 1 µg/ml for two days (Figure 6A, Table S2). We confirmed these results with real-time RT-PCR (Figure 6B), and further projected the functions and pathways of these genes using the Ingenuity Pathway Analysis (IPA). These genes were associated with more than 20 categories of biological functions above the threshold including neurological development/diseases, cardiovascular development, cell-to-cell signaling, skeletal disorder, and cell morphology (Figure 6C).

Of particular importance was that CDX1 induction caused up-regulation of several genes involved neuronal development including *Ntf3, Ednrα/β, Prom1 and Negr1* (Figure 6A-B & Table S2). In contrast, the expression levels of *Figf* (a gene important for vascular endothelial growth [22]), *c-Tnnt2*, and *Fgf8* (a cardiogenic regulator [23]), were markedly reduced (Figure 6A-B). In addition, consistent with our findings that CDX1 participated in epicardial EMT, the expression of cell adhesion molecules and genes involved in EMT (e.g., *Tcf21, Tbx18, Snai1, Dsc2, Cldn3/11/15, Chl1)*, as well as cadherin precursors was also altered upon CDX1 induction (Figure 6A-B & Table S2). We also identified the conserved binding sites of CDX1 at promoters of *Prom1* and *Figf* (Figure 6D). Luciferase reporter assays further revealed that CDX1 specifically activated *Prom1*, but significantly repressed transcript activity from the *Figf* promoter (Figure 6D). Although CDX1 was previously reported as an upstream regulator for *HOX* genes [5], [10], we did not observe significant alterations in transcript levels for most *HOX* family members (Figure S6A–C). These data thus supported the presence and function of non-*HOX* targets for CDX1 in epicardium.

## Discussion

Accumulating evidence suggests that epicardium and EPDCs play crucial roles in cardiac development [2], [3], [20]. Impairment of epicardial EMT or the migration and the maturation of EPDCs leads to a broad spectrum of cardiac disorders including coronary vascular abnormalities [2], [3], [20]. In this study, we demonstrated that CDX1 was temporarily expressed in hearts at 11.5 dpc, a time period that coincides with epicardial development. Our data further revealed that ectopic CDX1 expression enhanced epicardial EMT, whereas the high-dose induction or depletion of CDX1 blocked the migration and the differentiation of epicardium into vascular smooth muscle cells. Although induction of CDX1 *in vivo* in our transgenic mouse model might alter the function of different tissues (Figure S3), our observations on explanted hearts or epicardium isolated from embryos support the notion that the CDX1 acts cell autonomously, at least in part, during epicardial development.

CDX family members appear to function redundantly in development of a number of organs. For instance, we previously demonstrated that a more severe hematopoietic defect exists with compound *Cdx* deficiency than loss of function of any single *Cdx* gene causes [11]. Further, offsprings of double *Cdx1* and *Cdx2* knockout mice exhibit more pronounced vertebral homeosis than seen in either of the single mutant background [24], [25]. Although previous studies demonstrated that *Cdx1* and *Cdx4* double mutant mice were viable without notable cardiac defects reported [26], due to the embryonic lethality of trophectodermal defects in *Cdx2* deficiency [7], potential cardiac dysfunction has not been investigated with compound *Cdx* mutant background. We did not detect any *Cdx4* transcripts in epicardium, but the mRNA levels of *Cdx2* were higher than *Cdx1* in epicardium (data not shown). Our data suggest that CDX1 affects the migration and the differentiation of EPDCs into vascular smooth muscles. Nevertheless, ectopic expression of CDX1 appeared to result in more severe phenotype than CDX1 knockdown. It is conceivable that the transient deficiency of CDX1 in epicardium can be masked by compensation of other CDX family members, or alternatively, by some undefined mechanisms *in vivo* during embryonic development.

Multiple transcription factors such as SLUG, E-cadherin, TCF21, WT1, and TBX18 have been implicated in EMT and lineage development of EPDCs [2], [3], [20]. Among them, WT1 and TBX18 act as epicardial reporters [4], [27], whereas the inhibition of TCF21 leads to increased differentiation of EPDCs into vascular smooth muscles [28]. Additionally, the expression of SLUG is usually increased during EMT process with downregulation of E-cadherin. In our study, CDX1 induction, the expression of *Wt1, Tcf21, Tbx18*, and *E-cadherin* was reduced, whereas the levels of *Slug* were increased. Furthermore, *Figf*, a vascular endothelial growth factor, appeared to be directly downregulated upon ectopic expression of CDX1. These expression data thus support our findings that high-level CDX1 promotes epicardial EMT, but inhibits the formation of coronary arteries and the differentiation of EPDCs.

The typical epithelium consists of interconnected sheets with tight junctions and adherens, whereas mesenchymal cells lack these connections and adapt a stellate morphology that facilitates their movement and differentiation into a number of distinct cell types. Alterations of tight junctions and adherens are often associated with EMT processes in diverse developmental settings and in human diseases including carcinogenesis. We observed pronounced alteration in expression of many adhesion molecules such as *Dsc2, Cldn2/3/6/15/11*, and *Chl1* with RNA-seq analyses. Notably, in this regard, a number of studies report that CDX family members are involved in regulating tight junctions in intestinal epithelium and colorectal tumors [29], [30], [31], suggesting a functional conservation of CDX1 in EMT from various tissues.

CDX1 refines the positional identity of vertebrate hindbrain by repressing *MAFB* expression [32]. In addition, *Cdx* genes are transiently expressed in the caudal region of the neural plate, and act as important determinants for the rostrocaudal identity of neural progenitors [33]. In our RNA-seq analyses, we revealed that CDX1 induction upregulated the expression of genes in neuronal development including *Negr1, Ntf3, Nefm*, and *Ednrα/β*. By contrast, the expression levels of genes important for cardiac development (e.g., *Fgf8* and *C-Tnnt2*) were significantly decreased as well as *Figf*, a gene that plays a key role in angiogenesis. We thus propose a working model of CDX1 in epicardial function (Figure 6E). Transient high expression of CDX1 at 11.5 dpc promotes epicardial EMT. CDX1 does, however, need to be downregulated later in embryonic development to allow proper migration and maturation of EPDCs. According to our expression analyses, it is plausible that a high-level expression of CDX1 for a prolonged period may disrupt the differentiation of EPDCs by promoting expression of genes in neuronal lineages.

The use of EPDCs for cell therapy has displayed their positive effects in heart repair in combination with cardiac progenitors [2], [3], [20]. Research into various cell-autonomous capacities of adult epicardium may eventually lead to novel therapeutic applications. Therefore, it will be of great interest to explore whether CDX1 promotes the growth of vascular smooth muscle and enhances re-vascularization during the repair process of damaged hearts in the adult mice. Nevertheless, our current findings clearly illuminate a previously undefined role of CDX1 in epicardial development, and also improved our understanding of transcriptional network in the differentiation of EPDCs during embryogenesis.

## Supporting Information

Figure S1 Establishing doxycycline-inducible CDX1 transgenic ESCs and mouse model. (A) Real-time RT-PCR of CDX1 expression on two representative doxycycline-inducible CDX1 ESC lines with or without doxycycline treatment. (B) Karyotyping on a doxycycline-inducible CDX1 ESC line used for establishing transgenic mouse model. Fourteen out of sixteen randomly checked cells contains normal number of chromosomes as 40, with two other cells harboring 39 chromosomes. (C) Genotyping of embryos collected from pregnant C57BL/6 female mice crossed with *Cdx1^Tg^rtTA^+/+^* male mice. CDX1 primers were designed to cross introns and to detect CDX1 transgene at 265 bp. Wildtype (WT) ROSA26 locus was amplified at 350 bp and a fragment of 250 bp could be amplified when rtTA was inserted at this locus.
(PDF)

Figure S2 Low-dose leakage of CDX1 did not affect embryonic development. (A) Gross morphology of different organs isolated from *rtTA^+/−^* and *Cdx1^Tg^rtTA^+/−^* collected from embryos treated with 10 µg/ml doxycycline in drinking water starting from 11.5 dpc to 14.5 dpc. (B–C) WIHF with an antibody against PECAM on hearts isolated at 14.5 dpc from *rtTA^+/−^* and *Cdx1^Tg^rtTA^+/−^* embryos in the absence or presence of 10 µg/ml doxycycline for three days. (D) Gross morphology of *rtTA^+/−^* and *Cdx1^Tg^rtTA^+/−^* embryos collected at 14.5 dpc without doxycycline treatment. Photo of PCR results below shows the genotypes of the embryos. *Cdx1* transgene was amplified only in *Cdx1^Tg^rtTA^+/−^* embryos (eg. 1^st^ set: 2, 3 & 4; 2^nd^ set: 1, 2&6).
(PDF)

Figure S3 Continuous CDX1 induction in adult mice led to damage of small intestine and liver. (A) Body weight was measured over time on *rtTA^+/−^* and *Cdx1^Tg^rtTA^+/−^* mice in the absence or presence with 500 µg/ml doxycycline in drinking water. CDX1 induction resulted in a rapid drop of body weight in *Cdx1^Tg^rtTA^+/−^* mice, which were died within two weeks. (B–F) H&E staining of tissue sections collected from intestine (B), liver (C), heart (D), lung (E), and kidney (F). Upon induction of CDX1 in *Cdx1^Tg^rtTA^+/−^* mice, blebbing in submucosa and accumulation of red blood cells in villi lamina propria at small intestine were observed (as indicated by red arrows). (C) Gross morphology of liver in CDX1 induced mice demonstrated a overall pale appearance. Condensed nuclei were observed in the liver sections, indicating apoptosis of hepatocytes upon continuous CDX1 expression. Scale bars: 120 µm.
(PDF)

Figure S4 Ectopic expression of CDX1 affected epicardial development during embryogenesis. (A) IHF with antibodies against c-TNNT2 or WT1 on cardiac sections from *rtTA^+/−^* and *Cdx1^Tg^rtTA^+/−^* embryos at 14.5 dpc without any doxycycline treatment. (B) Establishing primary epicardial culture from embryonic heart at 11.5 dpc. (C) Confirmation of the epicardial origin of cultured cells with WT1, an epicardium-specific marker. (D) DAPI and WT1 staining on primary epicardium collected from *rtTA^+/−^* and *Cdx1^Tg^rtTA^+/−^* embryos at 14.5 dpc without any doxycycline treatment. (E) Invasion of CFMDA-labeled EPDCs into subepicardial layer of explanted hearts collected at 11.5 dpc was monitored at 0 h or 48 h post labeling in the presence of 1 µg/ml doxycycline. (F–G) BrdU incorporation assay on control epicardium or epicardium upon CDX1 induction in the presence of doxycycline (F). The percentage of BrdU-labeled cells was comparable in the two groups with or without CDX1 induction and summarized as bar graph below the panel (G).
(PDF)

Figure S5 *Cdx* genes in epicardium. (A–B) Real-time RT-PCR on *Cdx1* expression in primary epicardium which were collected from embryos at 11.5 dpc and cultured for 3 or 6 days *in vitro* (A), or in epicardium which were collected from embryos at 11.5 dpc and cultured in the absence or presence of 5 ng/ml TGF-β1 for three days (B). (C) Migration distance (initial distance was subtracted by the distance at the end of experiments) of primary epicardium collected from embryos at 11.5 dpc with CDX1 induction by doxycycline at two different doses or with *Cdx1* knockdown compared to controls. (D) Quantification of α-SMA+ cells in primary epicardium collected from embryos at 11.5 dpc with CDX1 induction or with *Cdx1* knockdown compared to controls. (C–D): **: *p*<0.01. (E) Real-time RT-PCR on *Cdx2* expression in primary epicardium upon CDX1 induction or knockdown. (F) Real-time RT-PCR on *Cdx1* expression in primary epicardium which were collected from embryos at 11.5 dpc or in whole hearts and cultured in the presence of two different doses of doxycycline treatment, relative to non-inducible controls.
(PDF)

Figure S6 Expression analyses in epicardium upon ectopic induction of CDX1 compared to non-inducible control cells. (A–C) Expression of *HOX* gene clusters was analyzed by real-time RT-PCR in epicardium upon ectopic induction of CDX1 compared to non-inducible control cells. (D) Example of network in neuronal development affected by CDX1 induction in epicardium. (E) Example of network in cell adhesion affected by CDX1 induction in epicardium. (D–E): Proteins in red color: up-regulated upon CDX1 induction; Proteins in green color: downregulated upon CDX1 induction.
(PDF)

Table S1 Primers used for RT-PCR.
(XLSX)

Table S2 Genes altered upon CDX1 induction in epicardium.
(XLSX)

## References

[1] Vincent SD, Buckingham ME (2010) How to make a heart: the origin and regulation of cardiac progenitor cells. Curr Top Dev Biol 90: 1–41. 20691846 10.1016/S0070-2153(10)90001-X

[2] Gittenberger-de Groot AC, Winter EM, Bartelings MM, Goumans MJ, DeRuiter MC, et al (2012) The arterial and cardiac epicardium in development, disease and repair. Differentiation 84: 41–53. 22652098 10.1016/j.diff.2012.05.002

[3] von Gise A, Pu WT (2012) Endocardial and epicardial epithelial to mesenchymal transitions in heart development and disease. Circ Res 110: 1628–1645. 22679138 10.1161/CIRCRESAHA.111.259960 PMC3427736

[4] Martinez-Estrada OM, Lettice LA, Essafi A, Guadix JA, Slight J, et al (2010) Wt1 is required for cardiovascular progenitor cell formation through transcriptional control of Snail and E-cadherin. Nat Genet 42: 89–93. 20023660 10.1038/ng.494 PMC2799392

[5] Young T, Deschamps J (2009) Hox, Cdx, and anteroposterior patterning in the mouse embryo. Curr Top Dev Biol 88: 235–255. 19651307 10.1016/S0070-2153(09)88008-3

[6] Davidson AJ, Ernst P, Wang Y, Dekens MP, Kingsley PD, et al (2003) cdx4 mutants fail to specify blood progenitors and can be rescued by multiple hox genes. Nature 425: 300–306. 13679919 10.1038/nature01973

[7] Deb K, Sivaguru M, Yong HY, Roberts RM (2006) Cdx2 gene expression and trophectoderm lineage specification in mouse embryos. Science 311: 992–996. 16484492 10.1126/science.1120925

[8] Savory JG, Mansfield M, Rijli FM, Lohnes D (2011) Cdx mediates neural tube closure through transcriptional regulation of the planar cell polarity gene Ptk7. Development 138: 1361–1370. 21350009 10.1242/dev.056622

[9] Silberg DG, Swain GP, Suh ER, Traber PG (2000) Cdx1 and cdx2 expression during intestinal development. Gastroenterology 119: 961–971. 11040183 10.1053/gast.2000.18142

[10] Subramanian V, Meyer BI, Gruss P (1995) Disruption of the murine homeobox gene Cdx1 affects axial skeletal identities by altering the mesodermal expression domains of Hox genes. Cell 83: 641–653. 7585967 10.1016/0092-8674(95)90104-3

[11] Wang Y, Yabuuchi A, McKinney-Freeman S, Ducharme DM, Ray MK, et al (2008) Cdx gene deficiency compromises embryonic hematopoiesis in the mouse. Proc Natl Acad Sci U S A 105: 7756–7761. 18511567 10.1073/pnas.0708951105 PMC2409377

[12] Paik EJ, Mahony S, White RM, Price EN, Dibiase A, et al (2013) A Cdx4-Sall4 Regulatory Module Controls the Transition from Mesoderm Formation to Embryonic Hematopoiesis. Stem Cell Reports 1: 425–436. 24286030 10.1016/j.stemcr.2013.10.001 PMC3841246

[13] Savory JG, Bouchard N, Pierre V, Rijli FM, De Repentigny Y, et al (2009) Cdx2 regulation of posterior development through non-Hox targets. Development 136: 4099–4110. 19906845 10.1242/dev.041582

[14] Bai YQ, Miyake S, Iwai T, Yuasa Y (2003) CDX2, a homeobox transcription factor, upregulates transcription of the p21/WAF1/CIP1 gene. Oncogene 22: 7942–7949. 12970742 10.1038/sj.onc.1206634

[15] Lengerke C, Wingert R, Beeretz M, Grauer M, Schmidt AG, et al (2011) Interactions between Cdx genes and retinoic acid modulate early cardiogenesis. Dev Biol 354: 134–142. 21466798 10.1016/j.ydbio.2011.03.027 PMC3502019

[16] Beard C, Hochedlinger K, Plath K, Wutz A, Jaenisch R (2006) Efficient method to generate single-copy transgenic mice by site-specific integration in embryonic stem cells. Genesis 44: 23–28. 16400644 10.1002/gene.20180

[17] Wang Y, Yates F, Naveiras O, Ernst P, Daley GQ (2005) Embryonic stem cell-derived hematopoietic stem cells. Proc Natl Acad Sci U S A 102: 19081–19086. 16357205 10.1073/pnas.0506127102 PMC1323159

[18] Zhong S, Joung JG, Zheng Y, Chen YR, Liu B, et al (2011) High-throughput illumina strand-specific RNA sequencing library preparation. Cold Spring Harb Protoc 2011: 940–949. 21807852 10.1101/pdb.prot5652

[19] Wang Q, Liu X, Tang N, Archambeault DR, Li J, et al (2013) GASZ promotes germ cell derivation from embryonic stem cells. Stem Cell Res 11: 845–860. 23816659 10.1016/j.scr.2013.05.012

[20] Lie-Venema H, van den Akker NM, Bax NA, Winter EM, Maas S, et al (2007) Origin, fate, and function of epicardium-derived cells (EPDCs) in normal and abnormal cardiac development. Scientific World Journal 7: 1777–1798. 18040540 10.1100/tsw.2007.294 PMC5901302

[21] Compton LA, Potash DA, Mundell NA, Barnett JV (2006) Transforming growth factor-beta induces loss of epithelial character and smooth muscle cell differentiation in epicardial cells. Dev Dyn 235: 82–93. 16258965 10.1002/dvdy.20629

[22] Avantaggiato V, Orlandini M, Acampora D, Oliviero S, Simeone A (1998) Embryonic expression pattern of the murine figf gene, a growth factor belonging to platelet-derived growth factor/vascular endothelial growth factor family. Mech Dev 73: 221–224. 9622638 10.1016/s0925-4773(98)00049-5

[23] Watanabe Y, Miyagawa-Tomita S, Vincent SD, Kelly RG, Moon AM, et al (2010) Role of mesodermal FGF8 and FGF10 overlaps in the development of the arterial pole of the heart and pharyngeal arch arteries. Circ Res 106: 495–503. 20035084 10.1161/CIRCRESAHA.109.201665 PMC2843098

[24] Savory JG, Pilon N, Grainger S, Sylvestre JR, Beland M, et al (2009) Cdx1 and Cdx2 are functionally equivalent in vertebral patterning. Dev Biol 330: 114–122. 19328777 10.1016/j.ydbio.2009.03.016

[25] van den Akker E, Forlani S, Chawengsaksophak K, de Graaff W, Beck F, et al (2002) Cdx1 and Cdx2 have overlapping functions in anteroposterior patterning and posterior axis elongation. Development 129: 2181–2193. 11959827 10.1242/dev.129.9.2181

[26] van Nes J, de Graaff W, Lebrin F, Gerhard M, Beck F, et al (2006) The Cdx4 mutation affects axial development and reveals an essential role of Cdx genes in the ontogenesis of the placental labyrinth in mice. Development 133: 419–428. 16396910 10.1242/dev.02216

[27] Christoffels VM, Grieskamp T, Norden J, Mommersteeg MT, Rudat C, et al (2009) Tbx18 and the fate of epicardial progenitors. Nature 458: E8–9 discussion E9–10. 19369973 10.1038/nature07916

[28] Braitsch CM, Combs MD, Quaggin SE, Yutzey KE (2012) Pod1/Tcf21 is regulated by retinoic acid signaling and inhibits differentiation of epicardium-derived cells into smooth muscle in the developing heart. Dev Biol 368: 345–357. 22687751 10.1016/j.ydbio.2012.06.002 PMC3414197

[29] Bhat AA, Sharma A, Pope J, Krishnan M, Washington MK, et al (2012) Caudal homeobox protein Cdx-2 cooperates with Wnt pathway to regulate claudin-1 expression in colon cancer cells. PLoS One 7: e37174. 22719836 10.1371/journal.pone.0037174 PMC3376107

[30] Funakoshi S, Ezaki T, Kong J, Guo RJ, Lynch JP (2008) Repression of the desmocollin 2 gene expression in human colon cancer cells is relieved by the homeodomain transcription factors Cdx1 and Cdx2. Mol Cancer Res 6: 1478–1490. 18819935 10.1158/1541-7786.MCR-07-2161

[31] Keller MS, Ezaki T, Guo RJ, Lynch JP (2004) Cdx1 or Cdx2 expression activates E-cadherin-mediated cell-cell adhesion and compaction in human COLO 205 cells. Am J Physiol Gastrointest Liver Physiol 287: G104–114. 14977637 10.1152/ajpgi.00484.2003

[32] Sturgeon K, Kaneko T, Biemann M, Gauthier A, Chawengsaksophak K, et al (2011) Cdx1 refines positional identity of the vertebrate hindbrain by directly repressing Mafb expression. Development 138: 65–74. 21098558 10.1242/dev.058727

[33] Nordstrom U, Maier E, Jessell TM, Edlund T (2006) An early role for WNT signaling in specifying neural patterns of Cdx and Hox gene expression and motor neuron subtype identity. PLoS Biol 4: e252. 16895440 10.1371/journal.pbio.0040252 PMC1502144

