# Supplementary figures and images for: A Novel Role of CDX1 in Embryonic Epicardial Development
Source: PLoS One. 2014 Jul 28;9(7):e103271. doi: 10.1371/journal.pone.0103271 (PMC4113346; doi:10.1371/journal.pone.0103271)

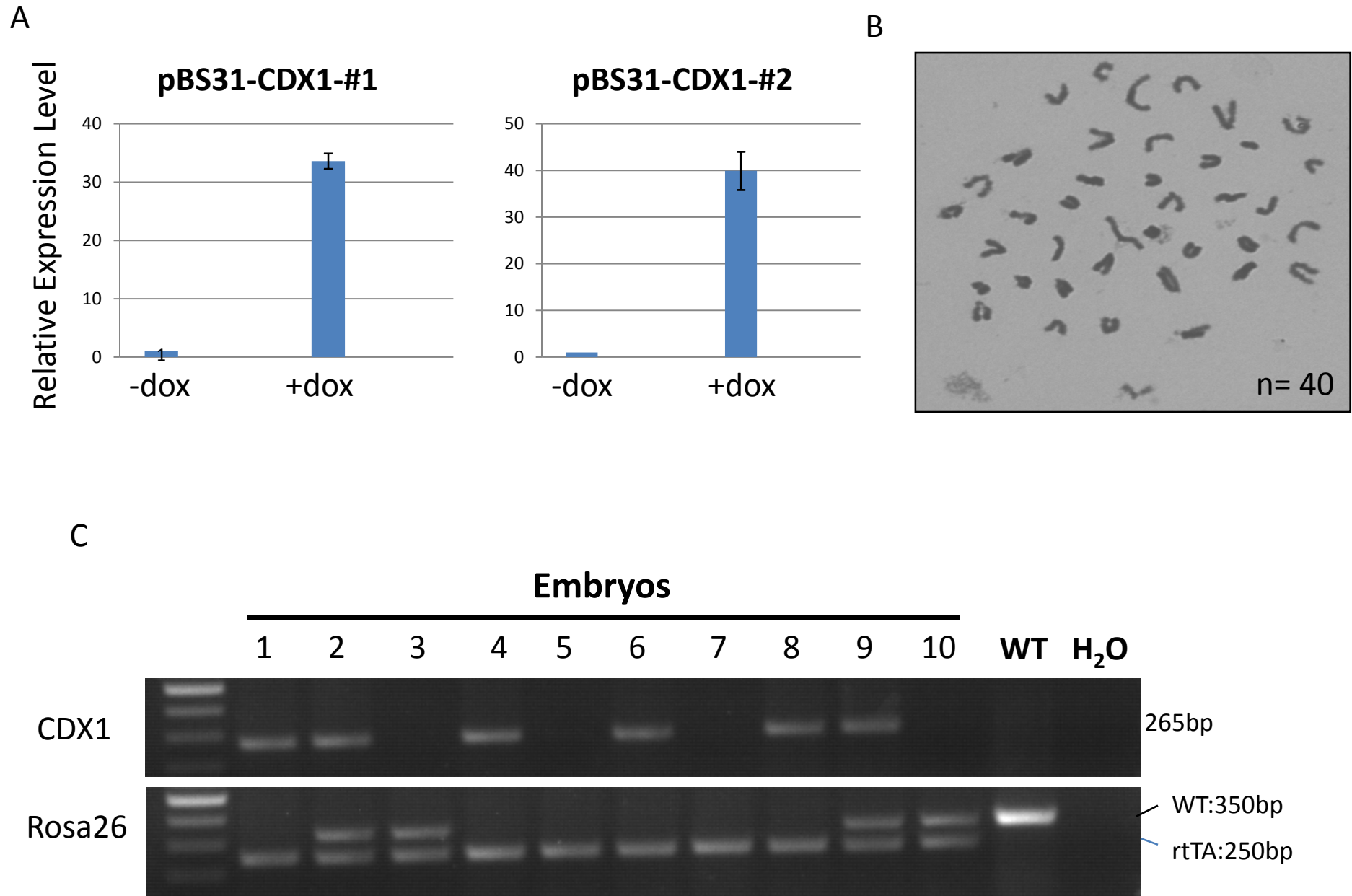

Supplement: Figure S1 — Establishing doxycycline-inducible CDX1 transgenic ESCs and mouse model. (A) Real-time RT-PCR of CDX1 expression on two representative doxycycline-inducible CDX1 ESC lines with or without doxycycline treatment. (B) Karyotyping on a doxycycline-inducible CDX1 ESC line used for establishing transgenic mouse model. Fourteen out of sixteen randomly checked cells contains normal number of chromosomes as 40, with two other cells harboring 39 chromosomes. (C) Genotyping of embryos collected from pregnant C57BL/6 female mice crossed with Cdx1TgrtTA+/+ male mice. CDX1 primers were designed to cross introns and to detect CDX1 transgene at 265 bp. Wildtype (WT) ROSA26 locus was amplified at 350 bp and a fragment of 250 bp could be amplified when rtTA was inserted at this locus. (PDF) [file pone.0103271.s001.pdf]

A

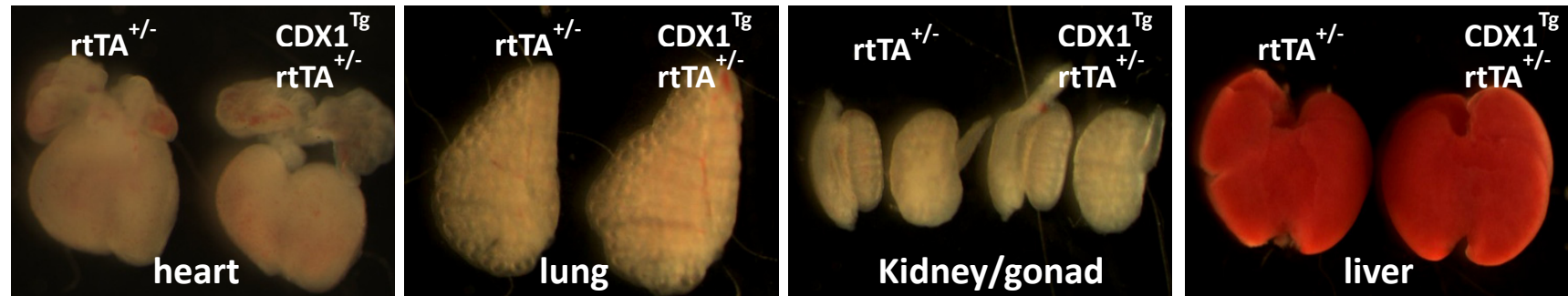

B

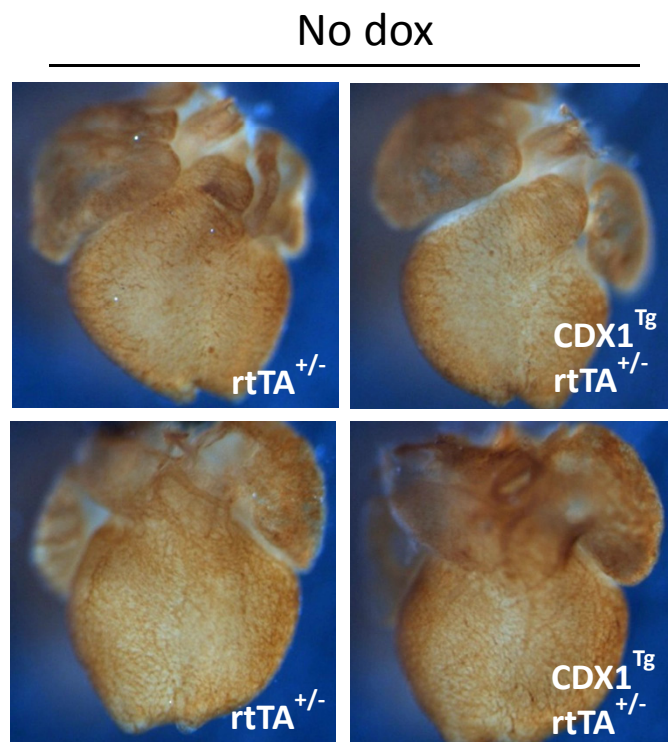

C

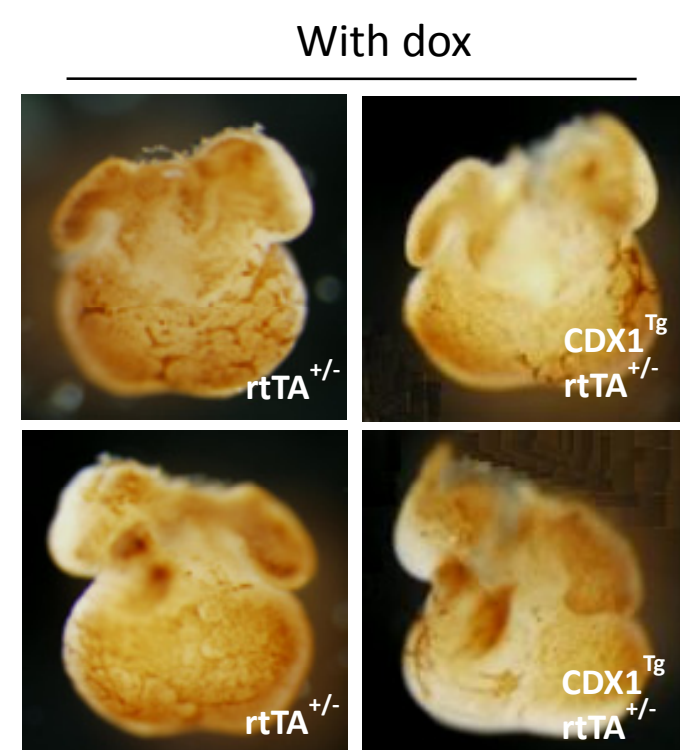

D

1st

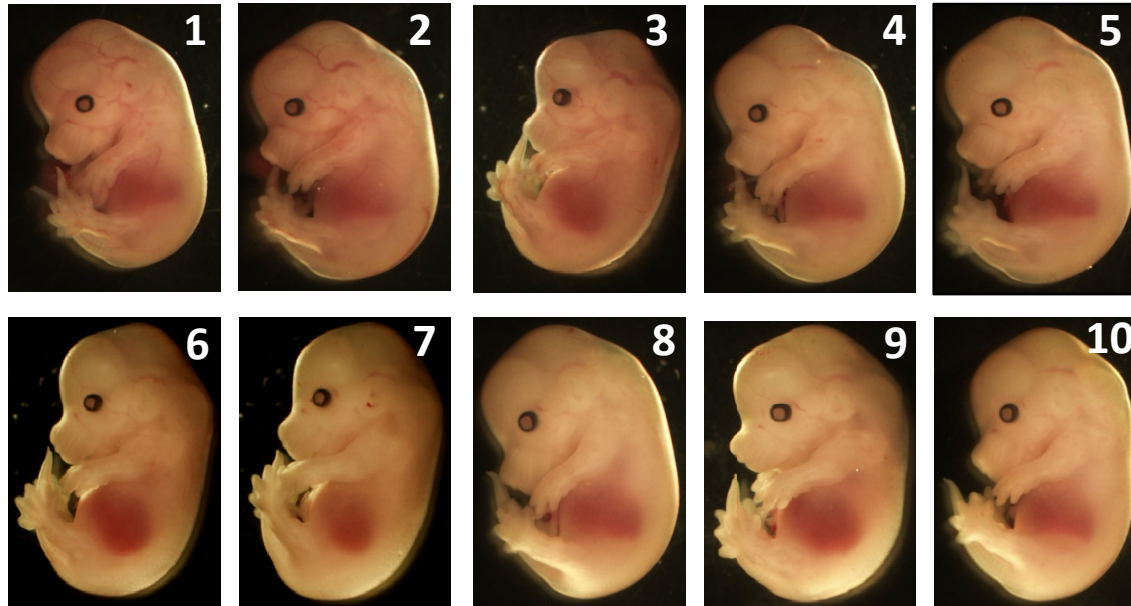

2nd

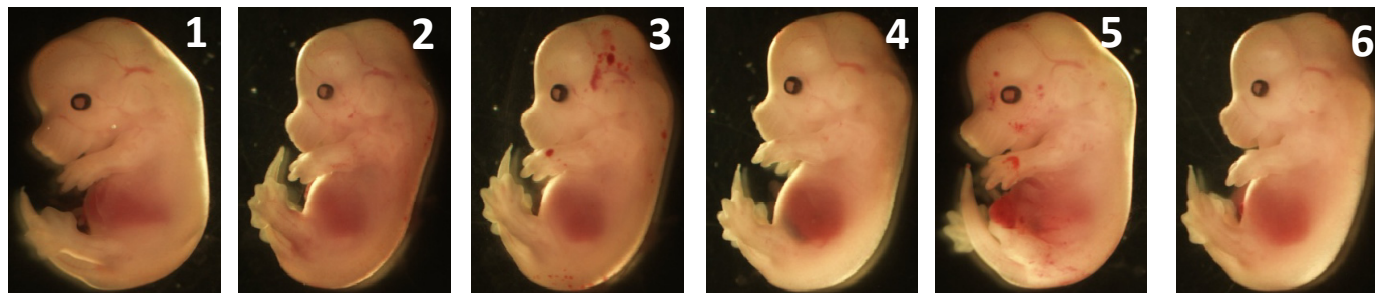

1

2

3

4

5

6

7

8

9

10

1

2

3

4

5

6

1<sup>st</sup> set of embryos

2<sup>nd</sup> set of embryos

Supplement: Figure S2 — Low-dose leakage of CDX1 did not affect embryonic development. (A) Gross morphology of different organs isolated from rtTA+/− and Cdx1TgrtTA+/− collected from embryos treated with 10 µg/ml doxycycline in drinking water starting from 11.5 dpc to 14.5 dpc. (B–C) WIHF with an antibody against PECAM on hearts isolated at 14.5 dpc from rtTA+/− and Cdx1TgrtTA+/− embryos in the absence or presence of 10 µg/ml doxycycline for three days. (D) Gross morphology of rtTA+/− and Cdx1TgrtTA+/− embryos collected at 14.5 dpc without doxycycline treatment. Photo of PCR results below shows the genotypes of the embryos. Cdx1 transgene was amplified only in Cdx1TgrtTA+/− embryos (eg. 1st set: 2, 3 & 4; 2nd set: 1, 2&6). (PDF) [file pone.0103271.s002.pdf]

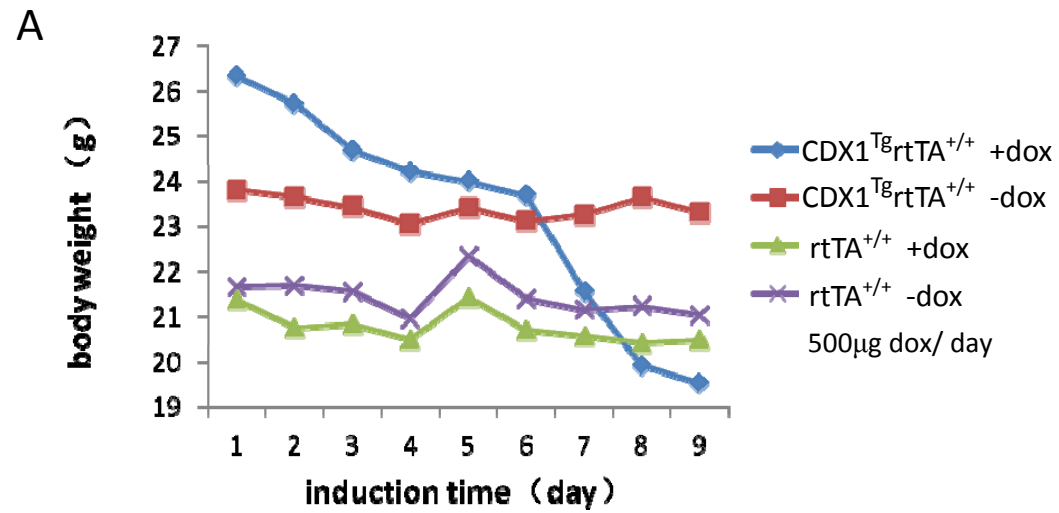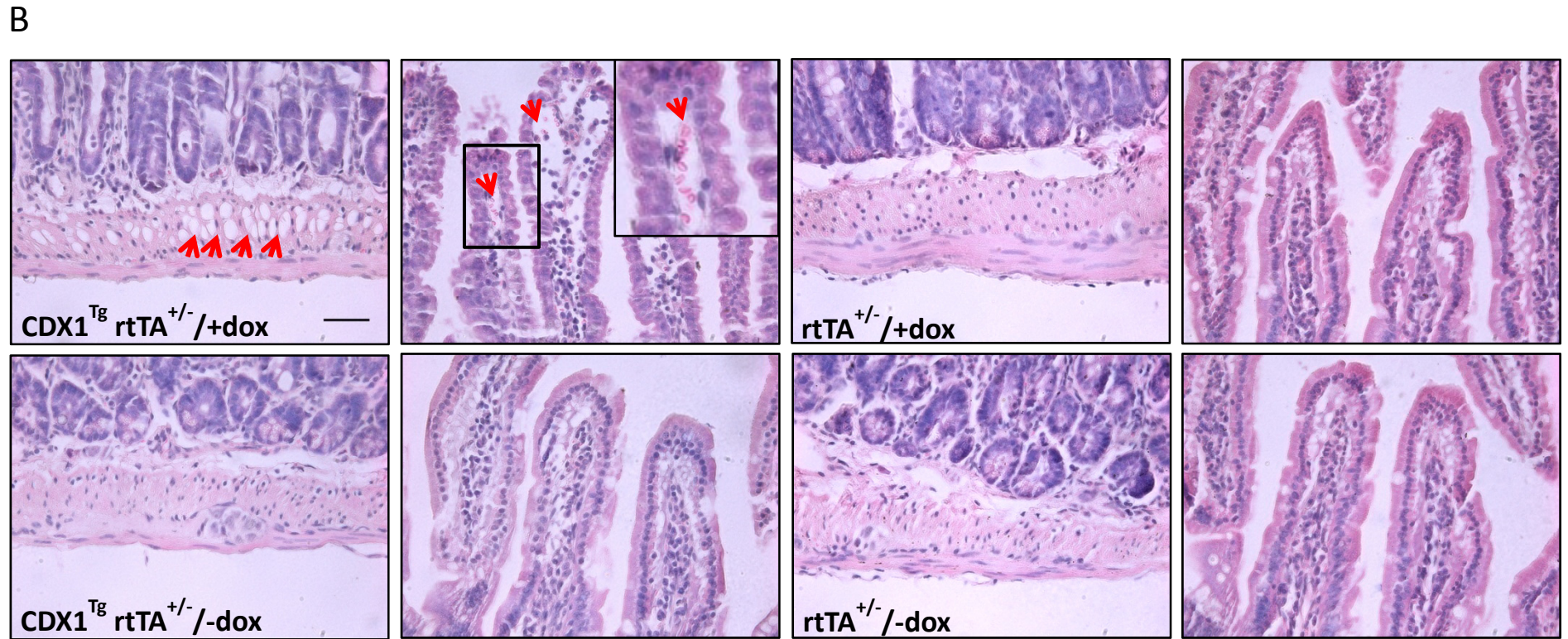

C

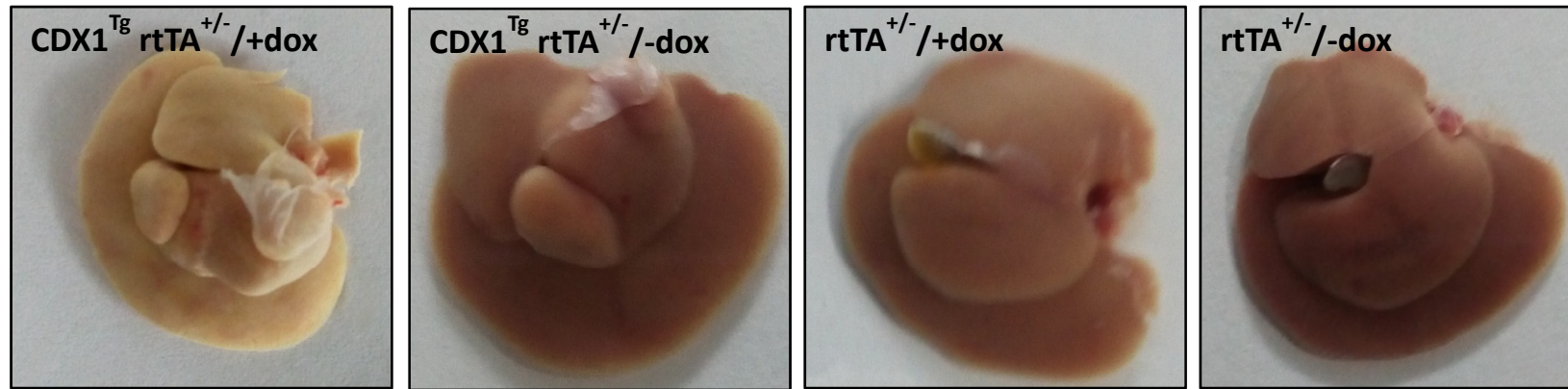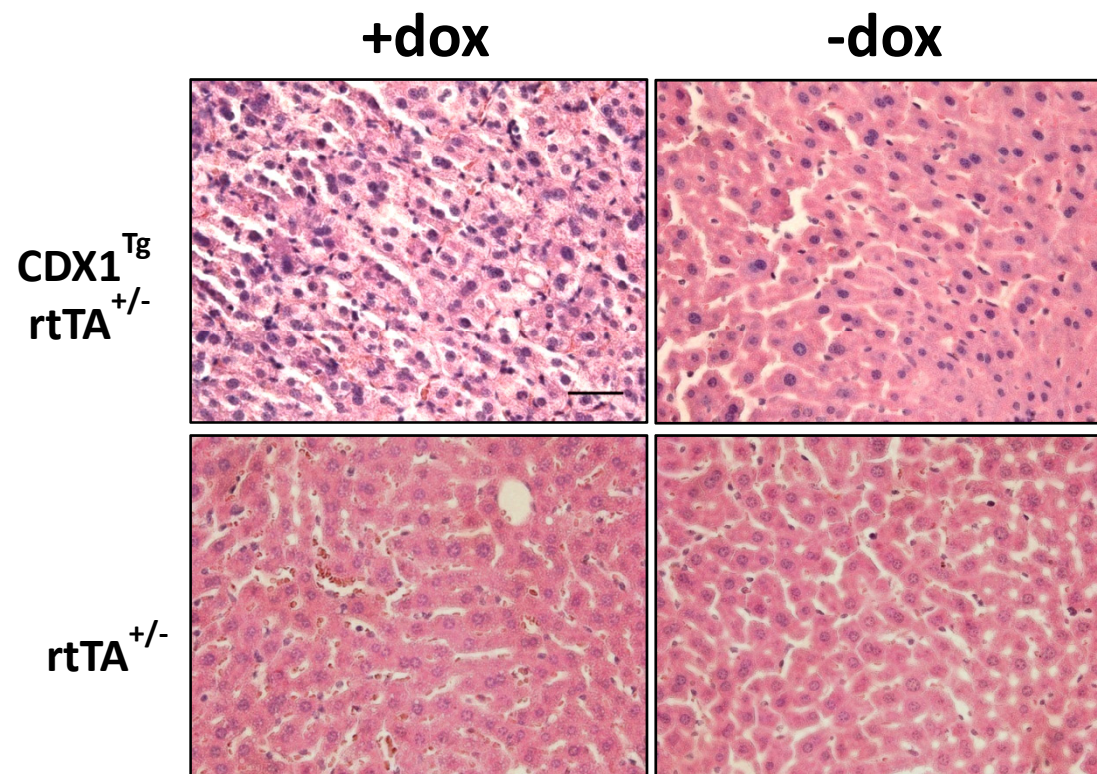

D heart

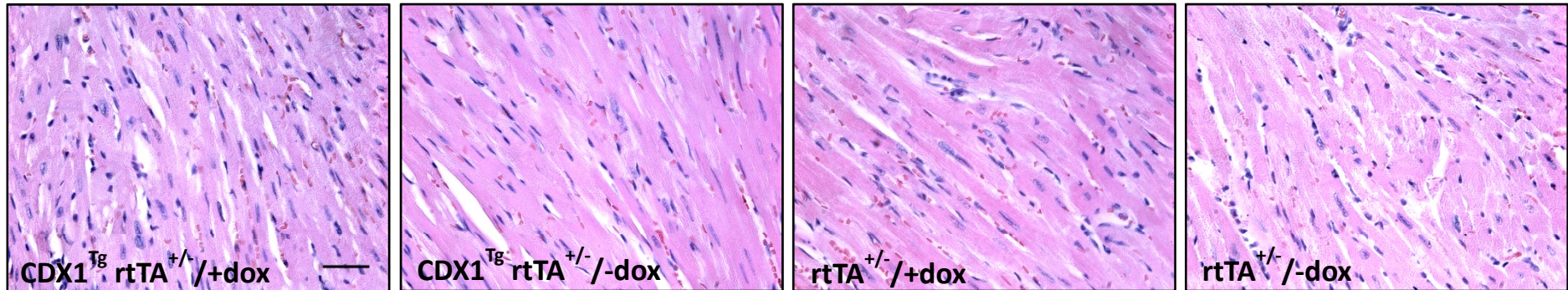

E Lung

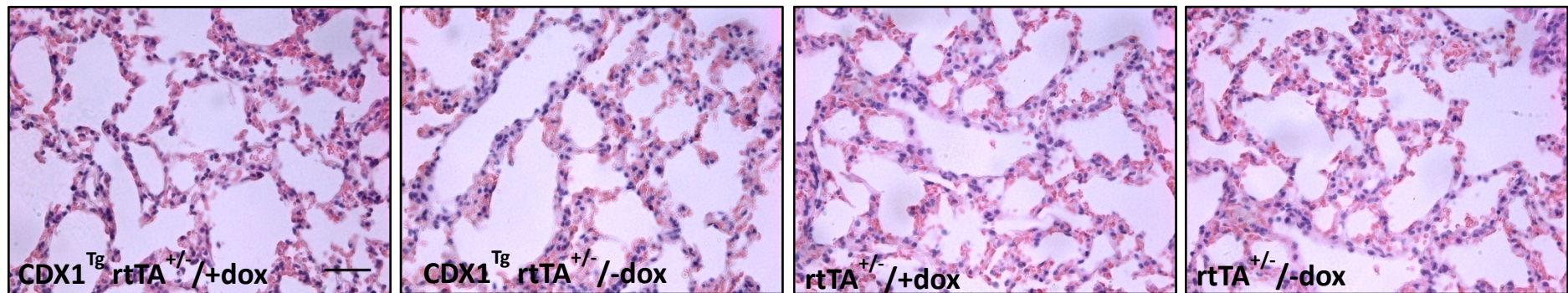

F kidney

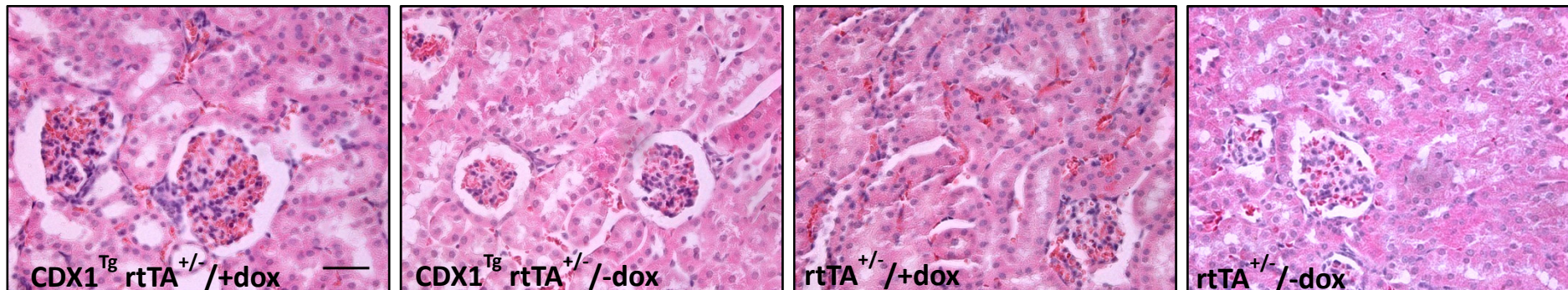

Supplement: Figure S3 — Continuous CDX1 induction in adult mice led to damage of small intestine and liver. (A) Body weight was measured over time on rtTA+/− and Cdx1TgrtTA+/− mice in the absence or presence with 500 µg/ml doxycycline in drinking water. CDX1 induction resulted in a rapid drop of body weight in Cdx1TgrtTA+/− mice, which were died within two weeks. (B–F) H&E staining of tissue sections collected from intestine (B), liver (C), heart (D), lung (E), and kidney (F). Upon induction of CDX1 in Cdx1TgrtTA+/− mice, blebbing in submucosa and accumulation of red blood cells in villi lamina propria at small intestine were observed (as indicated by red arrows). (C) Gross morphology of liver in CDX1 induced mice demonstrated a overall pale appearance. Condensed nuclei were observed in the liver sections, indicating apoptosis of hepatocytes upon continuous CDX1 expression. Scale bars: 120 µm. (PDF) [file pone.0103271.s003.pdf]

A

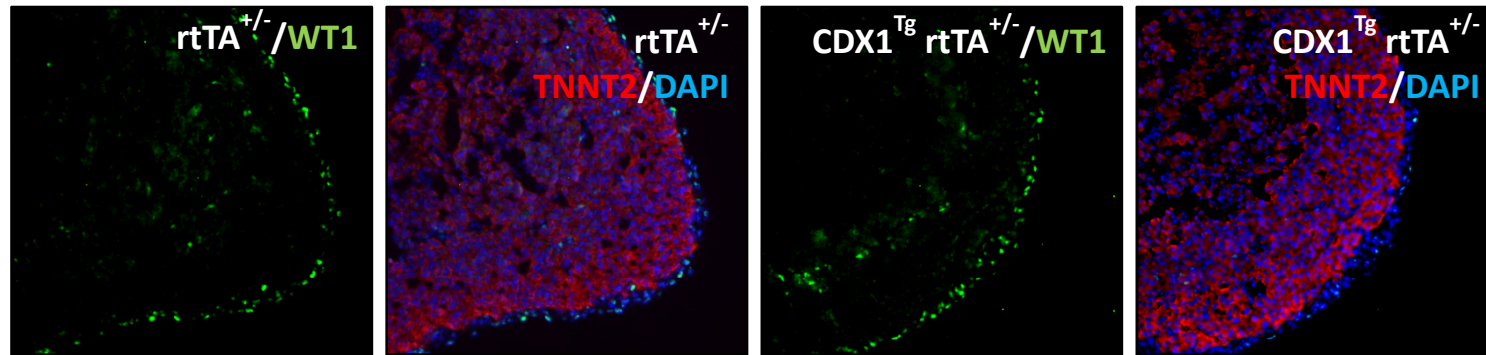

B

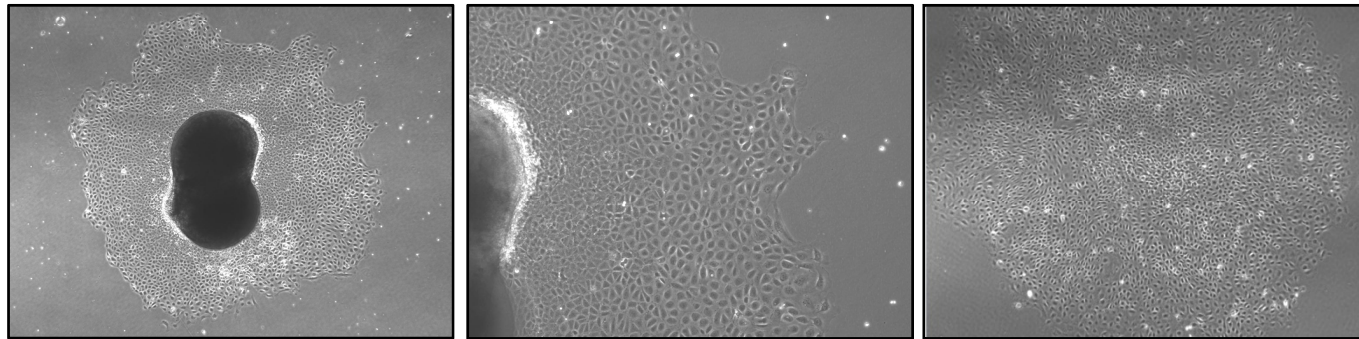

C

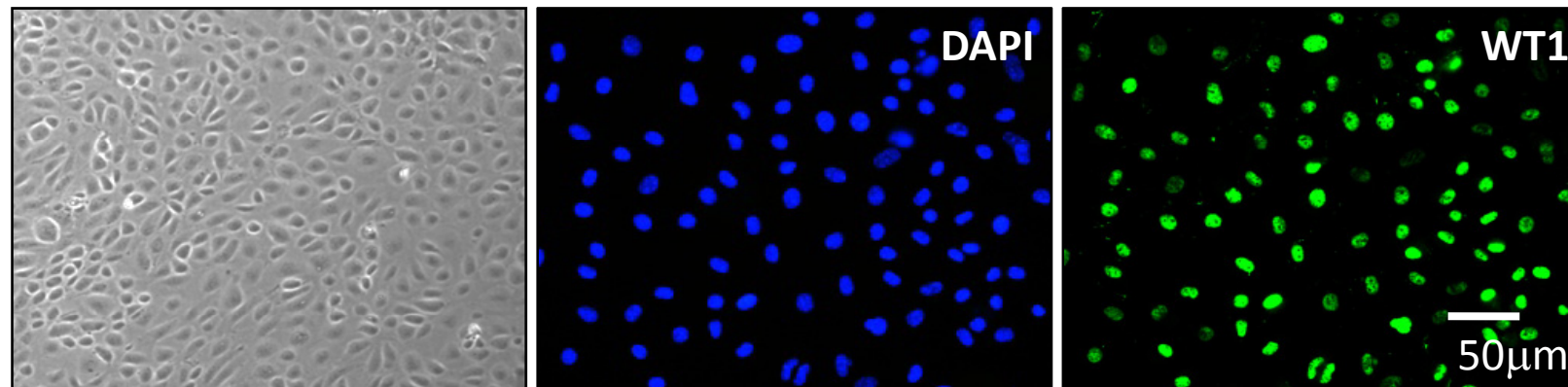

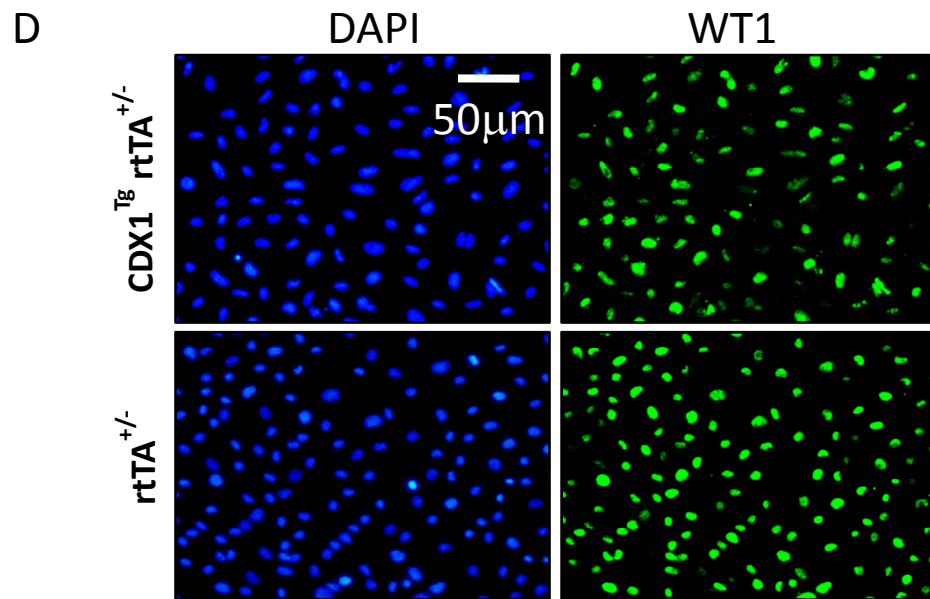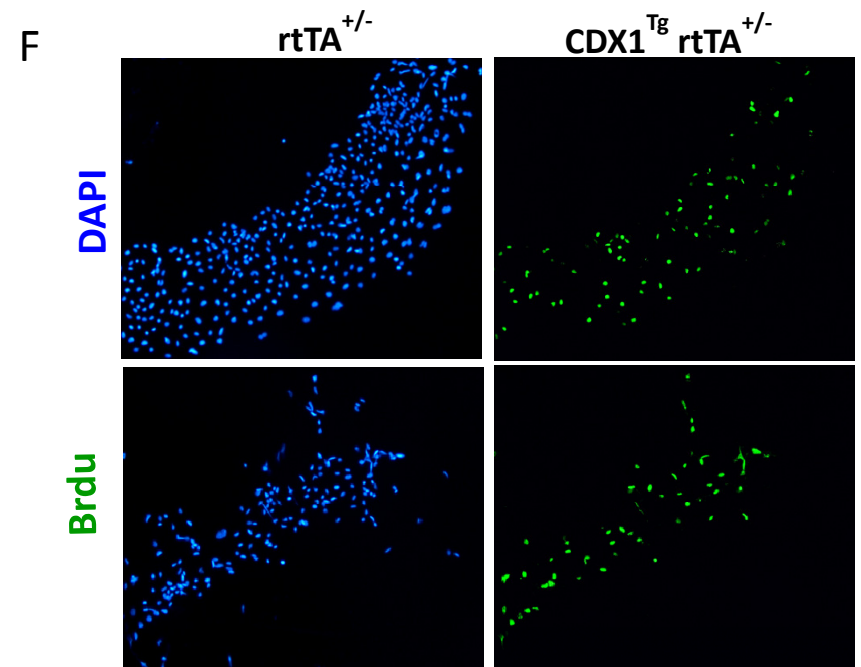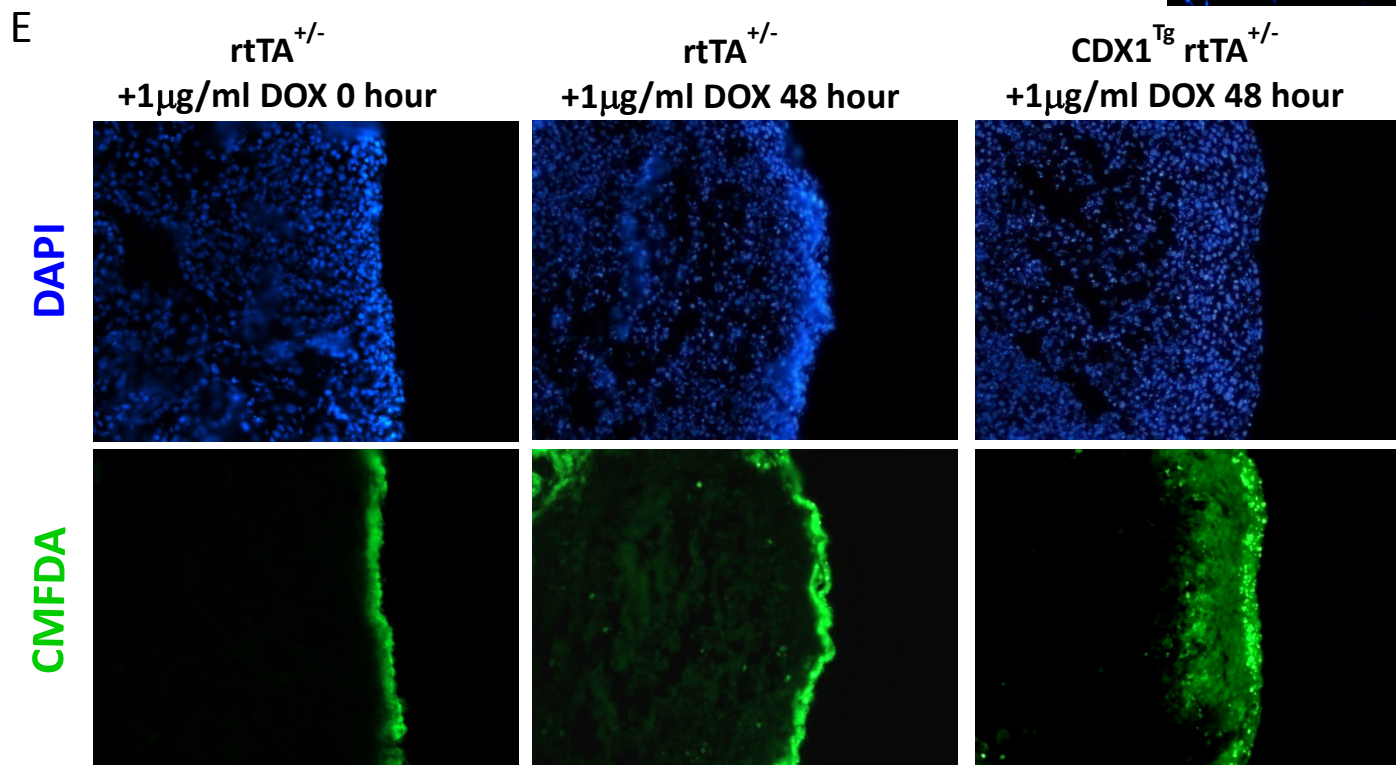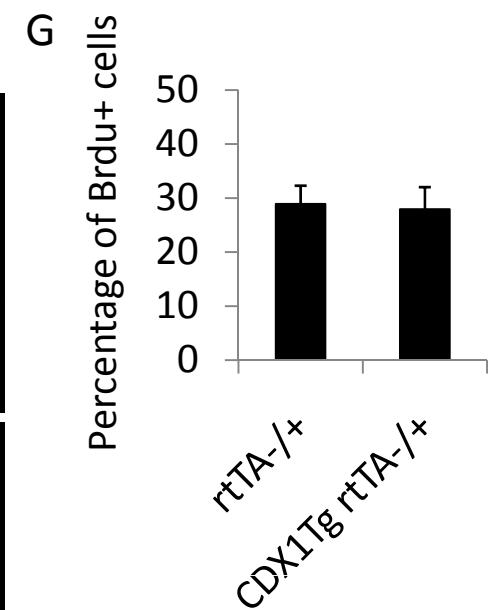

Chu et al.,  
Supporting Figure 4

Supplement: Figure S4 — Ectopic expression of CDX1 affected epicardial development during embryogenesis. (A) IHF with antibodies against c-TNNT2 or WT1 on cardiac sections from rtTA+/− and Cdx1TgrtTA+/− embryos at 14.5 dpc without any doxycycline treatment. (B) Establishing primary epicardial culture from embryonic heart at 11.5 dpc. (C) Confirmation of the epicardial origin of cultured cells with WT1, an epicardium-specific marker. (D) DAPI and WT1 staining on primary epicardium collected from rtTA+/− and Cdx1TgrtTA+/− embryos at 14.5 dpc without any doxycycline treatment. (E) Invasion of CFMDA-labeled EPDCs into subepicardial layer of explanted hearts collected at 11.5 dpc was monitored at 0 h or 48 h post labeling in the presence of 1 µg/ml doxycycline. (F–G) BrdU incorporation assay on control epicardium or epicardium upon CDX1 induction in the presence of doxycycline (F). The percentage of BrdU-labeled cells was comparable in the two groups with or without CDX1 induction and summarized as bar graph below the panel (G). (PDF) [file pone.0103271.s004.pdf]

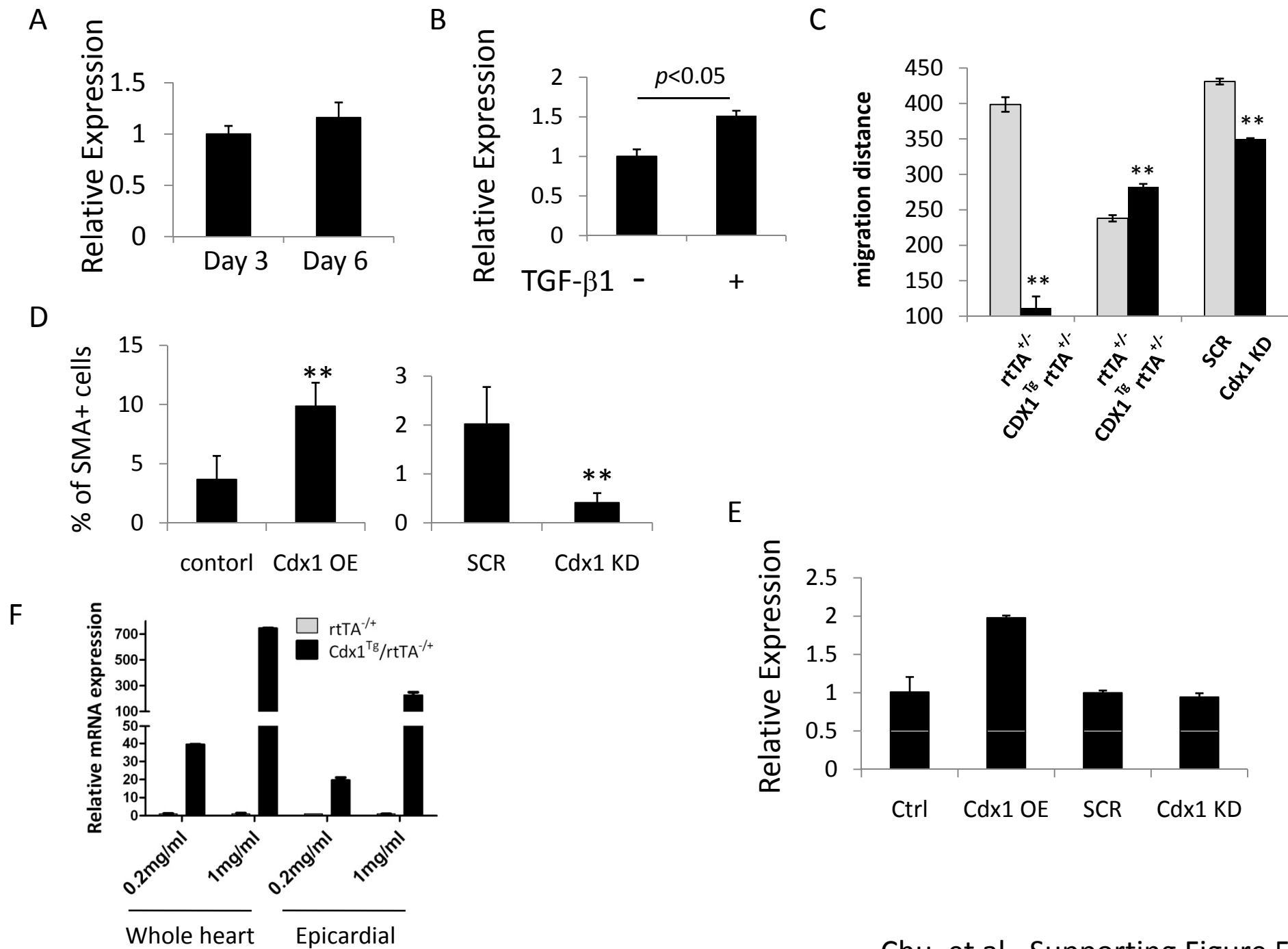

Supplement: Figure S5 — Cdx genes in epicardium. (A–B) Real-time RT-PCR on Cdx1 expression in primary epicardium which were collected from embryos at 11.5 dpc and cultured for 3 or 6 days in vitro (A), or in epicardium which were collected from embryos at 11.5 dpc and cultured in the absence or presence of 5 ng/ml TGF-β1 for three days (B). (C) Migration distance (initial distance was subtracted by the distance at the end of experiments) of primary epicardium collected from embryos at 11.5 dpc with CDX1 induction by doxycycline at two different doses or with Cdx1 knockdown compared to controls. (D) Quantification of α-SMA+ cells in primary epicardium collected from embryos at 11.5 dpc with CDX1 induction or with Cdx1 knockdown compared to controls. (C–D): **: p<0.01. (E) Real-time RT-PCR on Cdx2 expression in primary epicardium upon CDX1 induction or knockdown. (F) Real-time RT-PCR on Cdx1 expression in primary epicardium which were collected from embryos at 11.5 dpc or in whole hearts and cultured in the presence of two different doses of doxycycline treatment, relative to non-inducible controls. (PDF) [file pone.0103271.s005.pdf]

A

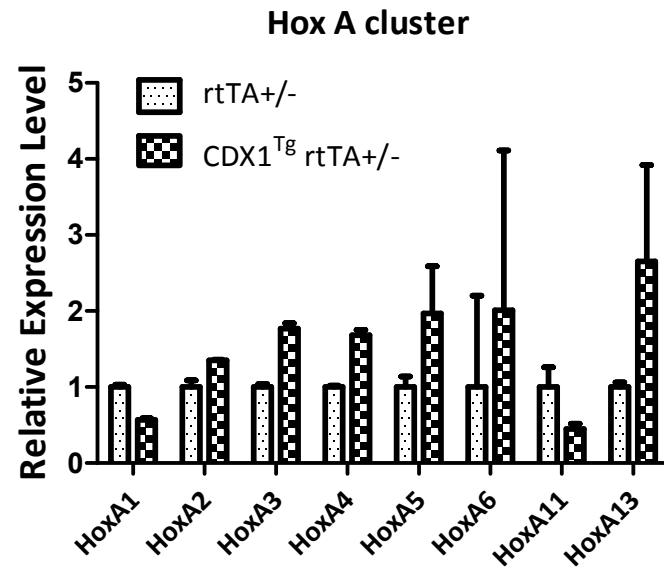

B

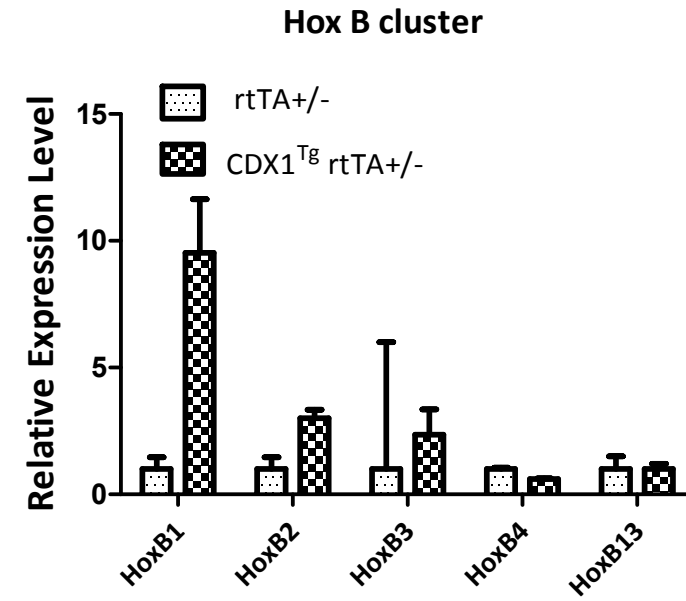

C

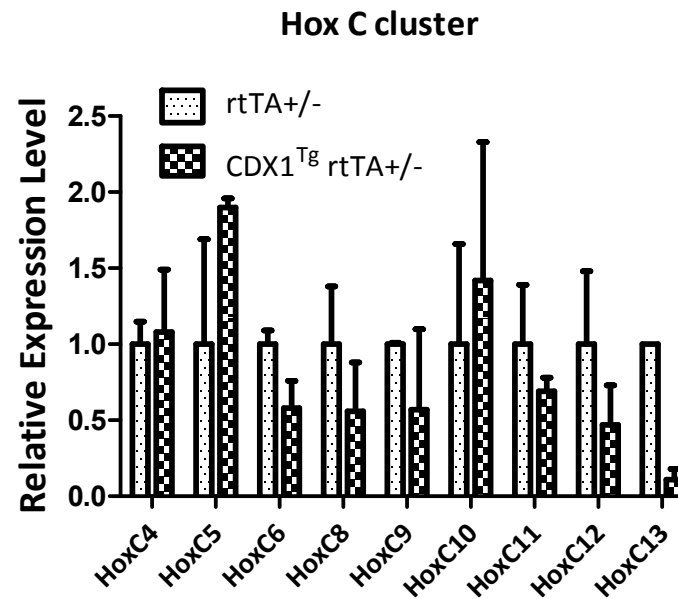

D

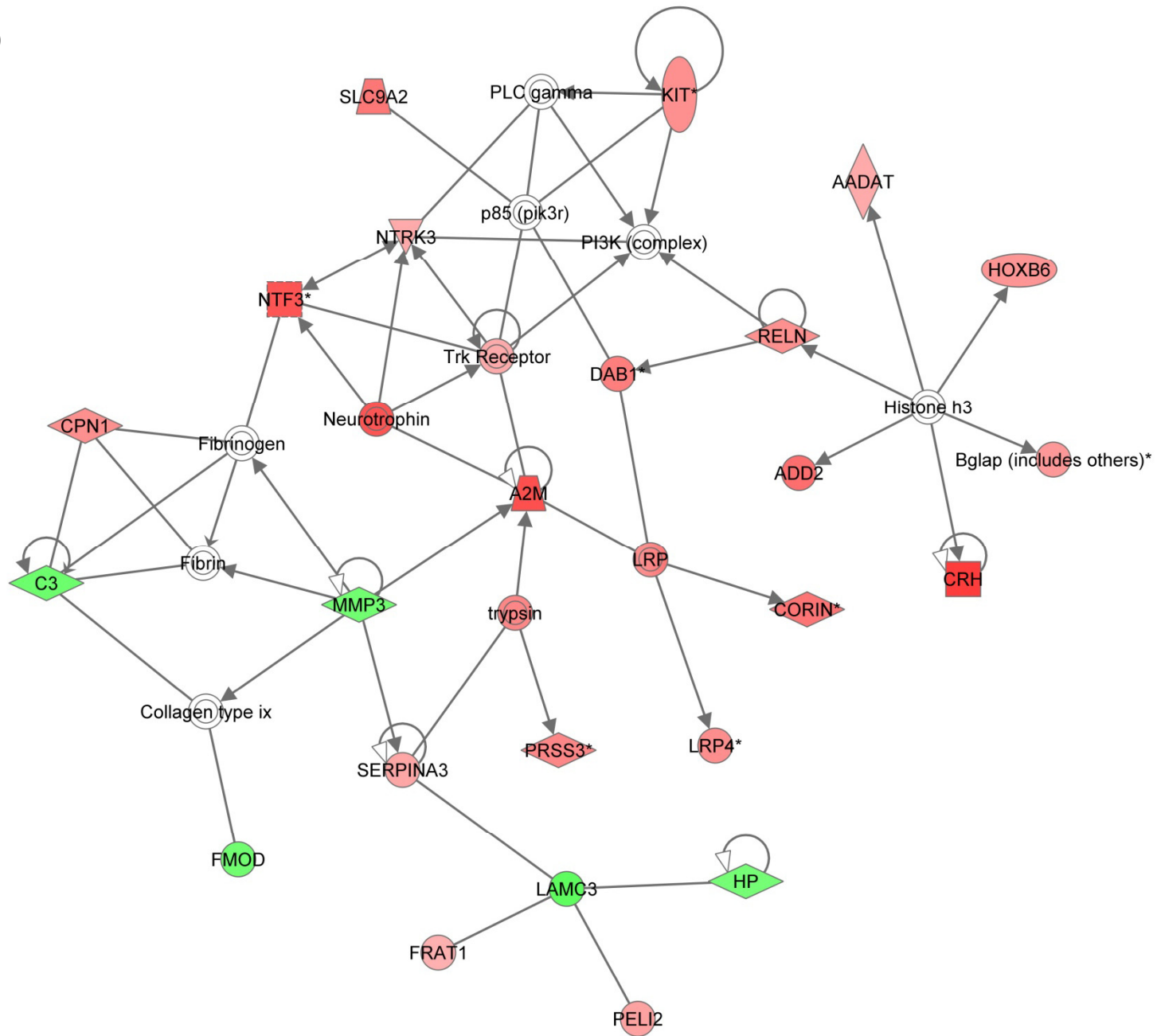

E

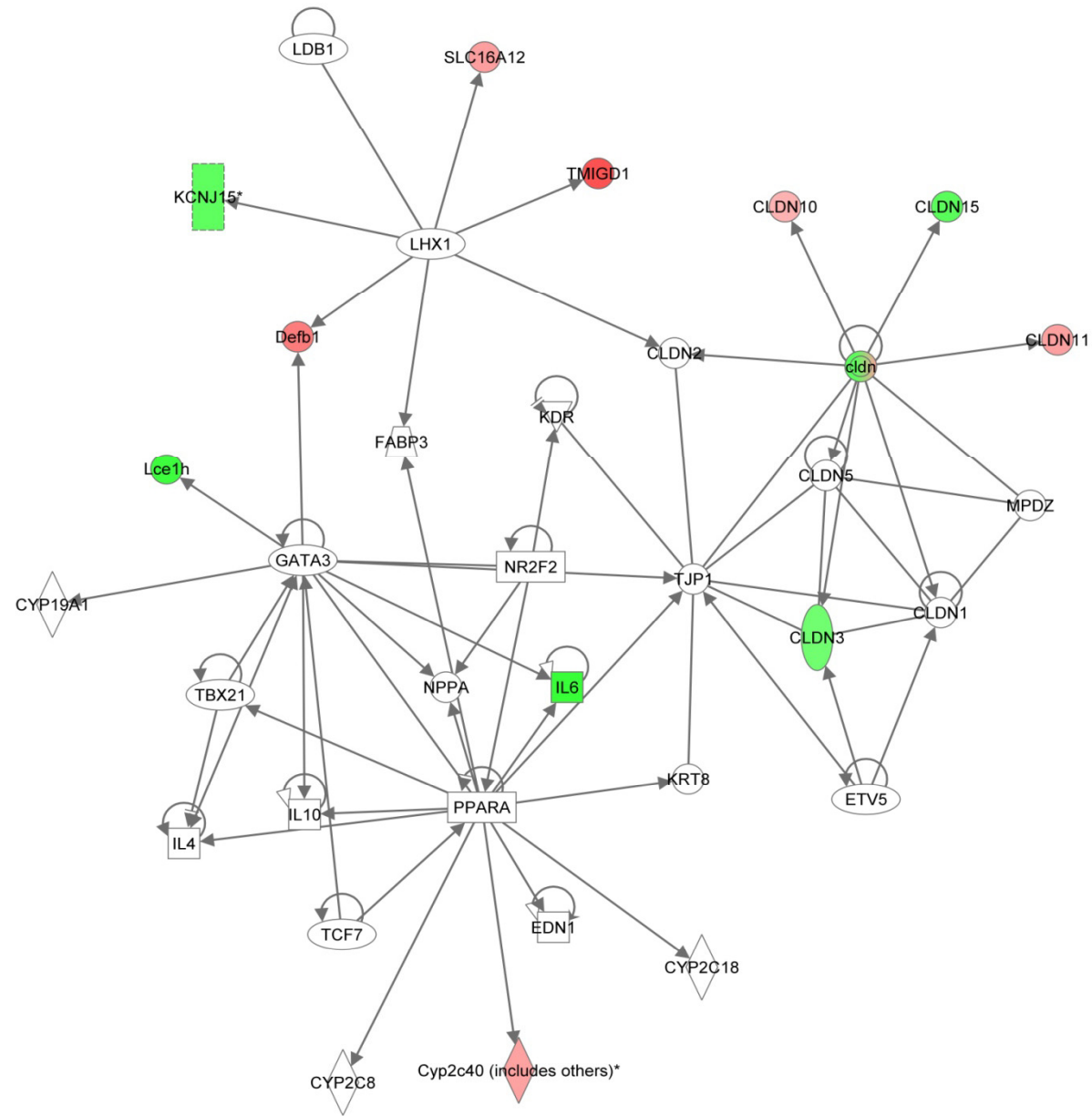

Supplement: Figure S6 — Expression analyses in epicardium upon ectopic induction of CDX1 compared to non-inducible control cells. (A–C) Expression of HOX gene clusters was analyzed by real-time RT-PCR in epicardium upon ectopic induction of CDX1 compared to non-inducible control cells. (D) Example of network in neuronal development affected by CDX1 induction in epicardium. (E) Example of network in cell adhesion affected by CDX1 induction in epicardium. (D–E): Proteins in red color: up-regulated upon CDX1 induction; Proteins in green color: downregulated upon CDX1 induction. (PDF) [file pone.0103271.s006.pdf]
